# Using BioPAX-Parser (BiP) to enrich lists of genes or proteins with pathway data

**DOI:** 10.1186/s12859-021-04297-z

**Published:** 2021-09-30

**Authors:** Giuseppe Agapito, Mario Cannataro

**Affiliations:** 1 Department of Legal, Economic and Social Sciences, University “Magna Graecia”, Catanzaro, Italy; 2 Department of Medical and Surgical Sciences, University “Magna Graecia”, Catanzaro, Italy; 3 Data Analytics Research Center, University “Magna Graecia”, Catanzaro, Italy

**Keywords:** Biological pathway, Statistical analysis, Pathway enrichment analysis, Pathway databases

## Abstract

**Background:**

Pathway enrichment analysis (PEA) is a well-established methodology for interpreting a list of genes and proteins of interest related to a condition under investigation. This paper aims to extend our previous work in which we introduced a preliminary comparative analysis of pathway enrichment analysis tools. We extended the earlier work by providing more case studies, comparing BiP enrichment performance with other well-known PEA software tools.

**Methods:**

PEA uses pathway information to discover connections between a list of genes and proteins as well as biological mechanisms, helping researchers to overcome the problem of explaining biological entity lists of interest disconnected from the biological context.

**Results:**

We compared the results of BiP with some existing pathway enrichment analysis tools comprising Centrality-based Pathway Enrichment, pathDIP, and Signaling Pathway Impact Analysis, considering three cancer types (colorectal, endometrial, and thyroid), for a total of six datasets (that is, two datasets per cancer type) obtained from the The Cancer Genome Atlas and Gene Expression Omnibus databases. We measured the similarities between the overlap of the enrichment results obtained using each couple of cancer datasets related to the same cancer.

**Conclusion:**

As a result, BiP identified some well-known pathways related to the investigated cancer type, validated by the available literature. We also used the Jaccard and meet-min indices to evaluate the stability and the similarity between the enrichment results obtained from each couple of cancer datasets. The obtained results show that BiP provides more stable enrichment results than other tools.

**Supplementary Information:**

The online version contains supplementary material available at 10.1186/s12859-021-04297-z.

## Background

Over the past decade, we have witnessed an increase in the production and availability of omics data aided by the continuous development and refinement of Next Generation Sequencing (NGS), Genome-Wide Association Studies (GWAS), gene expressions, and SNP microarrays platforms, also known as High-Throughput (HT) methodologies. Their ability to produce a massive amount of data has spurred the development of several pipelines for data analysis [1–4]. Several annotation software tools use Gene Ontology (GO) to link HT data analysis results with the affected biological mechanisms [5, 6]. Although these software tools can effectively analyze these vast amounts of available data, the produced results are still not connected to the biological mechanisms they may influence. Common diseases (for example, flu and headaches) and complex conditions (for example, cancer or diabetes) are due to several biological entities’ interactions represented through biological pathways.

Biological pathways are human representations of the existent biomolecules’ interactions regulating cellular functions in healthy and diseased conditions and how cells can interact with the external environment. Biological pathways are networks where nodes represent biomolecules, and edges represent the interaction among them. The pathway representation as a network has contributed to the development of several network alignment [7], and deep learning analysis [8] algorithms.

Biological pathways are fundamental in analyzing, contextualizing and interpreting omics data. The main pathway categories are three: Signalling Pathways, Metabolic Pathways and Regulatory Pathways. Several pathway databases including Kyoto Encyclopedia of Gene and Genome (KEGG) [9], Metabolic Pathway Database (MetaCyc) [10], PantherDB [11], PathwayCommons [12], Pathway Interaction Databse (PID) [13], Reactome [14], SIGnaling Network Open Resource (SIGNOR) [15] and WikiPathways [16] are available online. These databases share different types of pathways. For example, Reactome and KEGG store all three categories of pathways, while SIGNOR includes only signaling pathways and Metacyc showcases only metabolic pathways. Also, databases that contain the same kinds of pathways (like KEGG and Reactome) show minimal overlap on the number of pathways and gene coverage as reported in [17].

Many molecular and cell biologists face a common question in their research: how to link pathways to a specific genes or proteins list? The availability of biological information in a digital format enables the automatic elaboration of these pathway databases for different knowledge discovery tasks.

In particular, Pathway Enrichment Analysis (PEA) is a well-established approach to gain insight into the underlying biological mechanism of a differentially expressed list of genes and proteins of interest. PEA can use the information in pathway databases to elucidate the link between the genes and proteins of interest and the biological mechanisms affected in the biological pathways. The three principal categories of PEA methods are: (1) Over Represented Analysis (ORA); (2) Gene Set Enrichment Analysis (GSEA); and (3) Topological Enrichment Analysis (TEA).

The first two categories of methods perform enrichment analysis using a list of genes, proteins, SNPs or mRNA as input. Moreover, GSEA methods include self-contained or competitive null-hypothesis approaches. *Self-contained null hypothesis* methods assume that no genes in the gene list are associated with the phenotype. *Competitive null hypothesis* methods assume that genes in the gene list have a higher probability of being associated with the phenotype than genes outside the gene list. In contrast, the TEA methods need a genes and proteins list along with the network topology information.

PEA methods endeavor to help researchers decipher biological entities of interest disconnected from the biological context, expediting their findings’ validation. Analyzing lists of biological entities at the functional pathway-level can provide more explanatory power than analyzing a list of independent entities.

Several PEA software tools are available, among those: BioPAX-Parser (BiP) [18], Centrality-based Pathway Enrichment (CePa) [19], pathDIP [17] and Signaling Pathway Impact Analysis (SPIA) [20]. BiP and pathDIP belong to the ORA category. BiP employs *Hypergeometric* function [21] to assess the significance of genes of interest enriched in a specific pathway. Conversely, pathDIP uses a customized version of *Fisher’s Test* [22]. CePa and SPIA belong to the TEA category. CePa performs TEA by using network centrality measures (node input degree, node output degree, betweenness, input reachability and output reachability) [23]. In contrast, SPIA computes TEA using network measures [24] evaluating a node’s neighbor.

BiP and pathDIP can perform pathways enrichment analysis using a list of genes and proteins of interest without providing any additional information or data manipulation in the phenomena under investigation. In contrast, CePa and SPIA require users to provide additional network information, such as the interactions among the genes and proteins of interest and other network topology information, to perform TEA.

CePa, pathDIP, and SPIA allow performing PEA only using the previously collected and integrated pathway information. Conversely, BiP can perform PEA employing users’ downloaded pathways information from KEGG, Reactome or any other pathway database compliant with the Biological Pathway Exchange (BioPAX) format [25]. In this way, BiP can always provide more accurate and update enrichment results, avoiding losing critical biological features. Authors in [26] and [27] have remarkably highlighted the importance of using updated pathway information along with accurate lists of genes or proteins in performing pathway enrichment analysis. They proved that outdated pathway information negatively influenced the PEA results.

We evaluate the BiP’s pathway enrichment effectiveness using three TCGA cancer data sets and three GEO gene expression data sets, related respectively with colorectal (CC), endometrial (EC), and thyroid (TC) cancer. We used the six gene lists along with KEGG and Reactome pathway databases to evaluate the enrichment results. This way, it is possible to assess if the enrichment results obtained from each tool are similar, when varying the input gene list and keeping the database unchanged.

Although some works, such as [28, 29], use GO in PEA, as reported in Khatri et al. [30], the definition of pathway in certain cases may be misleading or incorrect. For this reason, we did not use GO to perform the experiments.

For each couple of cancer enrichment results we computed the pathway overlap (intersection) and the union between the two enrichments, selecting the first top 10 pathways (*p* value $$\le 0.005$$) to validate by using published literature. We chose as statistical significance threshold the *p* value$$ < 0.005$$, allowing us to improve the reproducibility of scientific studies as recommended in [31]. We used the *Jaccard* and *meet-min* similarities indices to evaluate the tools’ stability (for example, in terms of number and similarity of the enrichment pathways obtained by using different datasets related to the same cancer type). The obtained results proved that BiP is more solid in terms of number and similarity of enriched pathways than other existing tools. Also, BiP can identify some well-known cancer pathways omitted by other compared software frameworks.

This paper aims to extend our previous work [32] in which we introduced a preliminary comparative analysis of pathway enrichment analysis tools. In particular, we performed more case studies and compared BiP enrichment performance with other well-known PEA software tools.

## Methods

### Related works

PEA software frameworks are available as stand-alone software, web-based applications or program libraries. The first two categories are usually more convenient to use, as they do not require analytical skills or programming abilities. Program libraries are coded in C, Java, R and Python languages, allowing them to automate the process through scripting analysis pipelines. User skills and the cost-benefit ratio of time invested in orchestrating everything necessary to run the analysis may influence selecting software platforms and program libraries. In the following we present a description of some well-known PEA software frameworks.

- BiP [18] can perform PEA using pathways encoded in Biological Pathway Exchange (BioPAX) [25] and KGML (KEGG Markup Language) formats. BioPAX is a meta-language defined in OWL (Web Ontology Language) and represented in the RDF/XML (Resource Description Framework / eXtensible Meta Language) format and is the language of choice to store and exchange pathway data. KEGG Markup Language (KGML) is based on an XML-like markup language, providing computational analysis and modeling of gene/protein networks and chemical networks in the KEGG database. BiP has been extended to be compatible with the KGML format used to represent pathways in the KEGG database. BiP is developed in Java programming language, making it platform-independent. PEA in BiP is computed using a list of proteins/genes of interest as input. The genes and proteins enrichment is calculated using a customized version of *Hypergeometric Test*, along with multiple statistical corrector such as *False Discovery Rate* *(FDR)* and *Bonferroni*. BiP can enrich lists of genes and proteins using KEGG, Reactome and other available pathway databases compliant with the BioPAX format. BiP can be freely downloaded as a stand-alone application at [33].
- CePa [19] performs PEA based on topological information in addition to gene-set information. Pathways are collected and integrated from the Pathway Interaction Database (PID) database. PID includes the KEGG database. CePa parses the XML pathway files to obtain pathway data in order to perform the enrichment. To perform PEA, CePa needs a differentially expressed gene list and a background gene list. Several methods are available to produce the differential genes or proteins list, for example, the *t-test*. The background gene list is the list of genes of a specific microarray platform. The differential gene list and the background gene list must use the same identifiers to represent the gene symbol or protein identifier. CePa is an R application freely available at [34].
- pathDIP [17] is an integrated database of pathways in human, model organisms and domesticated animals, comprising core pathways from major curated pathway databases and gene pathway associations predicted using physical protein interactions. pathDIP helps researchers to perform ORA on structured ontology annotations, pathway databases, or set of biological entities. pathDIP is a web-application compatible with all available operating systems, it is available through an Application Program Interface (API) and it is developed in Java, R and Python. pathDIP computes PEA using the *Fisher’s Exact Test*, providing correction for multiple hypothesis testing through two different methods: *Bonferroni* and *FDR*. pathDIP integrates pathway data from 22 databases, including KEGG and Reactome. pathDIP is publicly available at [35].
- Signaling Pathway Impact Analysis (SPIA) [20] combines the evidence obtained from the classical enrichment analysis with the measure of the perturbation on a given pathway under a given condition. It allows to calculate a global pathway significance *p* value, combining the enrichment and perturbation *p* values. SPIA needs a set of differentially expressed genes and their fold changes and pathways topology to compute PEA from the condition under investigation. Differentially expressed genes need to be in Entrez gene IDs format. SPIA performs PEA by using pathway information from the KEGG database. SPIA is an R program freely available at [36].

The main differences among the surveyed PEA frameworks are summarised as follows. CePa and SPIA are all available as R packages, meaning the user needs some basic programming knowledge. Conversely, BiP efficiently performs PEA through a simple graphical user interface (GUI), that allows loading only a list of genes or proteins, selecting which pathway database use, setting the *p* value significance threshold and choosing the destination folder where to store the results. The only requirement to use BiP is to have previously installed Java, no additional libraries are necessary. pathDIP is a web application that allows performing enrichment analysis through a graphical interface just by loading a list of protein or gene identifiers and returning the pathways in which those identifiers are involved.

All the PEA software tools are different in terms of pathway enrichment calculation. BiP employs the Hypergeometric Test, along with FDR and Bonferroni correctors to calculate pathway enrichment. CePa computes pathway enrichment using topological information (that are, node input degree, node output degree, betweenness, input reachability and output reachability) and gene-set information. pathDip computes pathway enrichment using the *Fisher’s Exact Test*, along with *Bonferroni* and *FDR* correctors. Finally, SPIA calculates pathway enrichment combining the classical enrichment analysis with the measure of the perturbation on a given pathway under a given condition, evaluating a node’s neighbourhood.

BiP and pathDIP can perform pathways enrichment analysis using a list of genes and proteins of interest without providing any additional information or data manipulation in the phenomena under investigation. In contrast, CePa and SPIA require users to provide additional network information, such as the interactions among the genes and proteins of interest and other network topology information, to perform TEA.

Finally, BiP is the only enrichment analysis tool that allows users to select the pathway database that has to be used to enrich the condition under investigation.

All the PEA software tools and the related algorithms are well established in providing biological context in -omics studies, particularly to analyze the biological molecules, where summarizing the overall biology of a particular disease by pathways enhances interpretability. BiP, CePa, pathDIP and SPIA are some of the available gold standards tools in pathway enrichment analysis.

Most PEA tools evaluate enrichments in a very similar manner; we chose CePa, pathDip and SPIA as representative PEA tools due to their popularity and ability to test a breadth of data sources similar to that of BiP.

The Genomic Regions Enrichment of Annotations Tool (GREAT) [37] is a web-application for ontology enrichments tailored for regions bounding. GREAT requires a set of input genomic regions and an ontology of gene annotations as input, whereas the compared tools require as input a list of proteins or genes and one or more pathway databases. Also, ontology functional enrichment is different from PEA, how stated in [30]. Thus, we have chosen do not to compare GREAT with BiP.

g:Profiler [38] primary purpose is to perform functional enrichment analysis on input genes lists. Only in the last release of g:Profiler, pathway enrichment has been introduced, and it is limited only to Reactome and WikiPathways pathway databases. KEGG, due to licensing reasons, can be used only for terms association and not for enrichment. Conversely, from g:Profiler, all the compared PEA tools use KEGG to perform enrichment analysis, a not negligible difference that would make the comparison between the results obtained using different pathway databases unfair.

### Datasets

We downloaded three couples of cancer datasets: colorectal cancer (*COAD* [39] and *GSE41011* [40]), thyroid cancer (*THCA* [41] and *GSE65144* [42]), and endometrial cancer (*UCEC* [43], and *GSE63678* [44]) from the *TCGA* and *GEO* databases respectively, to test BiP.

The Gene Expression Omnibus (GEO) database is a public functional genomics database including high-throughput gene expression, chips, and microarrays data.

The Cancer Genome Atlas (TCGA) makes publicly available molecular and clinical information for more than 33 different types of human cancers, including exome (variant analysis), single nucleotide polymorphism (SNP), DNA methylation, transcriptome (mRNA), microRNA (miRNA) and proteome. TCGA data are accessible through the NCI Genomic Data Commons (GDC) data portal, GDC Legacy Archive, and the Broad Institute’s GDAC Firehose.

The COAD dataset contains 750 cases and 21, 224 probes, THCA includes 681 cases and 13, 564 probes, *UCEC* dataset comprises 560 cases and 22, 162 probes. The GSE41011 dataset contains 30, 968 probes and 19 cases, GSE65144 includes 12 cases and 54, 675 probes and, finally, GSE63678 comprises 7 cases and 22, 277 probes. Here cases indicate the positive samples, while probes indicate the dimension of the microarray (that is, the number of rows).

Table  1 summarises the information about the six dowloaded datasets.

**Table 1:** Summary of the downloaded datasets

Dataset Name	Data Source	Cancer Type	$\#$Cases	$\#$MGenes
COAD	TCGA	Colorectal Cancer (CC)	750	5913
THCA	TCGA	Thyroid Cancer (TC)	681	6270
UCEC	TCGA	Endometrial Cancer (EC)	560	6574
GSE41011	GEO	Colorectal Cancer (CC)	19	791
GSE65144	GEO	Thyroid Cancer (TC)	12	6202
GSE63678	GEO	Endometrial Cancer (EC)	7	1024

$\#MGenes$ refers to the number of mutated/deregulated genes involved with the various cancer types

We downloaded biological pathways database in BioPAX format for each available organism from Reactome [45], but we used Homo-sapiens to perform pathway enrichment analysis. Furthermore, we downloaded from KEGG [46] database all the Homo-sapiens pathways.

Reactome is an open-source freely available curated relational database of signaling and metabolic molecules and their relations organized into biological pathways and processes. Reactome uses pathway steps that indicate any event in biology that changes the state of a biological molecule. Molecules such as nucleic acids, proteins, complexes, and small molecules participating in reactions form a network of biological interactions called pathways.

KEGG is a database that integrates genomic, chemical and systemic functional information to analyze gene functions. KEGG contains various types of data classified as systems information, genomic information, chemical information and health information. The central KEGG element is the molecular network, representing systemic functions of the cell and the organism.

Mutated genes (MGenes) were downloaded from cBioPortal [47, 48] for the TCGA datasets (mutated + (Copy-number alterations) CNAs), while deregulated genes were obtained using the GEO2R web portal [49] for GEO datasets.

The cBioPortal for Cancer Genomics provides a Web resource for graphically analyzing multidimensional cancer genomics data. The portal reduces molecular profiling data from cancer tissues and cell lines into readily understandable genetic, gene expression, etc data. The cBioPortal allows to integrate multiple data types at the gene level and then query for the presence of specific biological events in each sample (for example, genetic mutation, gene amplification, and increased mRNA or miRNA expression). Data integration includes CNAs, mRNA and microRNA (miRNA) expression, and so on.

GEO2R is an online framework with which users can separate the samples into groups and select the differential expressed genes (DEG). We used the False Discovery Rate (FDR) corrector to adjust the *p* value by reducing the *type I* error in the null hypothesis due to multiple comparisons.

Both downloaded genes and pathways data sets have been used as input for the four PEA frameworks to get new biological insights.

We evaluated each tool’s capability to enrich the same relevant pathways using different data sets related to the same condition. Thus, it was not needed to perform any batch effect removal.

Additional file 1 contains the pathway enrichment analysis user guide illustrating how to download genes or proteins data sets from GEO and TCGA databses and highlighting how to use the downloaded data sets with the surveyed PEA software tools.

### Pathways enrichment computation

Equation 1 defines the *Hypergeometric* function $H(\cdot )$ implemented in BiP to compute the pathway enrichment score.

1 $$\begin{aligned} H(x)=\frac{\left( {\begin{array}{c}m\\ k\end{array}}\right) \left( {\begin{array}{c}n-m\\ n-k\end{array}}\right) }{\left( {\begin{array}{c}m\\ n\end{array}}\right) } \end{aligned}$$

In Eq. 1$``m''$ is the number of proteins into the pathway under enrichment analysis, $``n''$ refers to the number of proteins with which to perform the enrichment and $``k=m\cap n''`$ represents the intersection between the $``m''$ proteins in the pathway and the $``n''$ input proteins.

To balance the errors due to multiple tests, we implemented the False Discovery Rate (FDR) corrector along with the Bonferroni’s corrector. Equations  2 and  3 define FDR and Bonferroni correctors.

2 $$\begin{aligned} \alpha _{i}^{\prime }=\frac{p_i N}{i} \end{aligned}$$

In Eq. 2, $p_i$ is the *i*-th smallest *p* value out the total number of the *p* value for the performed experiment, *N* represents the number of performed tests, and *i* is the number of accepted *p* value using the *i*-th *p* value threshold.

3 $$\begin{aligned} \alpha ^{\prime }=\frac{\alpha }{k} \end{aligned}$$

In Eq.  3, $\alpha ^{\prime }$ is the corrected significance level, $\alpha$ is the chosen significance level, and *k* is the number of performed individual tests.

### Similarity indices computation

To assess the BiP abilities to detect essential pathways influenced by the genes under investigation, we compared the pathway enrichment results by using the two gene lists from the same disease obtained by BiP with respect to those provided by CePa, pathDIP, and SPIA, using the following similarity indices:

(1) **Jaccard similarity index** (*JI*) measures the percentage of similarity between the two enrichment sets. Equation  4 defines the Jaccard index.

4 $$\begin{aligned} JI = \frac{|P_1\cap P_2|}{|P_1\cup P_2|} \end{aligned}$$

In Eq.  4, $P_1$ and $P_2$ represent the total number of pathways in the two enrichments respectively. JI index range between 0 and 1, indicating respectively low and high similarity between the two enrichments.

(2) **meet-min index (mi)** [50] evaluates the similarity between the two sets, in terms of set containment. The definition of the *meet-min index* is reported in Eq. 5:

5 $$\begin{aligned} mi = \frac{|P_1\cap P_2|}{min(P_1, P_2)} \end{aligned}$$

In Eq. 5, $P_1$ and $P_2$ represent the total number of pathways respectively in the two enrichments sets. *mi index* ranges between 0, that indicates no containment between the two sets, and 1 that indicates a perfect containment of a set into another.

We chose to use Jaccard and meet-min indices as similarity measures for the following reasons. The Jaccard index is a common trend to compare populations by determining what percent of objects identified were present in both populations, which means that the Jaccard index evaluates the similarity of two sets. Meet-min is a measure that can assess the similarity (the common objects into the two sets). The containment between two sets or one set contains the other, which means that the meet-min can evaluate both similarity and containment between two sets.

## Results

This section compares the BiP, CePa, pathDIP and SPIA capability to perform pathway enrichment analysis using the six gene lists obtained from the cancer datasets in Table  1, using the Reactome and KEGG databases.

We compared BiP with pathDIP [17], CePa [19] and SPIA [20] based on the KEGG database. We also compared BiP and pathDIP using the Reactome database because CePa and SPIA do not support pathway data coming from this database. Then, we validated the identified pathways by using the available literature.

### Colorectal cancer enrichment using KEGG database

In this subsection, we present the pathway enrichment results obtained by all the software tools by analyzing the two gene lists related to Colorectal Cancer (CC) -*COAD* and *GSE41011*- and using the KEGG database.

BiP was able to identify 274 and 53 significant pathways (*p* value $\le 0.005$) respectively from the *COAD* and *GSE41011* genes lists (let see Additional file 2 for the whole enriched pathway list). Table  2 shows the intersection of the first top 10 (The first top ten pathways are the first ten ones ordered by *p* value from lower to higher values) pathways (*p* value $\le 0.005$) obtained from the two CC gene lists using the KEGG database.

**Table 2:** The intersection of the first 10 pathways (sorted by the relevance of *p* value $\le 0.005$) obtained from BiP by performing PEA using CC gene lists and the KEGG database

Pathway Name	*p* value	FDRc	Bc
(1) Metabolic pathways	4.33 × 10$^{-152}$	1.26 × 10$^{-149}$	1.26 × 10$^{-149}$
(2) Pathways in cancer	2.95 × 10$^{-36}$	2.14 × 10$^{-34}$	8.58 × 10$^{-34}$
(3) Transcriptional misregulation in cancer	2.44 × 10$^{-28}$	1.18 × 10$10^{-26}$	7.10 × 10$^{-26}$
(4) MAPK signaling pathway	2.04 × 10$^{-27}$	8.50 × 10$^{-26}$	5.95 × 10$^{-25}$
(5) Salmonella infection	2.75 × 10$^{-25}$	8.90 × 10$^{-24}$	8.01 × 10$^{-23}$
(6) Neuroactive ligand-receptor interaction	1.00 × 10$^{-24}$	2.65 × 10$^{-23}$	2.91 × 10$^{-22}$
(7) Herpes simplex virus 1 infection	1.85 × 10$^{-21}$	3.85 × 10$^{-20}$	5.39 × 10$^{-19}$
(8) Proteoglycans in cancer	3.08 × 10$^{-21}$	5.60 × 10$^{-20}$	8.95 × 10$^{-19}$
(9) Epstein-Barr virus infection	5.75 × 10$^{-21}$	9.29 × 10$^{-20}$	1.67 × 10$^{-18}$
(10) Human papillomavirus infection	1.48 × 10$^{-20}$	2.06 × 10$^{-19}$	4.32 × 10$^{-18}$

In the table, FDRc represents the corrected *p* value using FDR corrector, and Bc refers to the corrected *p* value using Bonferroni corrector

The first BiP’s enriched pathway is the *“Metabolic pathway”* that in a recent manuscript [51] is cited as pathway that regulates the colorectal cancer initiation and progression. In [52] authors identified how the *“Transcriptional misregulation in cancer”* pathway correlates with some outcomes of colorectal cancer. The *“MAPK signaling pathway”* regulates many cellular function including cell proliferation and apoptosis in colorectal cancer as reported in [53]. In [54] authors assessed the functional role of *“salmonella infection”* in downregulating *Wnt1* in the inflammatory response and colorectal cancer progression. The functional implication of *“Neuroactive ligand-receptor interaction pathway”* in colorectal cancer has been described in [55]. In [56] the implication of *“Herpes virus”* in human colorectal polyps and adenocarcinomas, inducing important oncogenic pathways in colon-cancer cells, is reported. In the manuscript published by Vicente et al. [57] the role of the *“proteoglycans molecules”* in colorectal cancer progression is described. In the review of Fernandes et al. [58] the role of *“Epstein-Barr virus infection”* and *“Human papillomavirus infection”* in the onset and progression of colorectal cancer, is assessed.

CePa was able to identify 24 and 22 significant pathways respectively from the *COAD* and *GSE41011* genes lists (let see Additional file 3 for the whole enriched pathway list).

Table  3 shows the CePa’s top 10 pathways obtained from the intersection of the pathway enrichment results for the two CC genes lists and the KEGG database.

CePa enrichment results include the following KEGG pathways. Authors show in [59] that Klotho family members are associated with *“FGFRs”* to adjust *“FGF”* binding to *“FGFRs”*, important molecules in CC [59, 60]. Satoh et al. [61] describes the importance of *“phyrymidine biosynthesis”* in colorectal cancer, while [62] highlights that loss of *“APC expression”* in tumor tissue may be related with the risk for recurrence and a poor survival rate for patients with colorectal cancer. Tsaniras et al. [63] reviews the link between pre-replicative complex and cancer (pathways *“Removal of licensing factors from origin”*, *“CDT1 association with the CDC6 ORC origin complex”*). Bernal et al. [64] evaluates the role of dysfunctional *“telomeres”* in contributing to genomic instability in cancer. Tong et al. [65] shows that *“ChREBP”* plays a critical role in redirecting glucose metabolism to anabolic pathways as well as suppressing p53 activity. Esteban-Jurado et al. [66] evaluates how the *“Fanconi anemia DNA damage repair pathway”* performs an important role in germline predisposition to colorectal cancer. Fernández-Briera et al. [67] describes how *“NCAM”* (but not the neurite outgrowth) is responsible of worse prognosis and lymph nodes metastasis in colorectal cancer. To the best of our knowledge, we could not find any evidence for the *“Switching of origins to a post-replicative state”* pathway.

**Table 3:** The intersection of the first 10 pathways obtained from CePa by performing pathway enrichment analysis by using CC genes lists and the KEGG database. The table summarises the values obtained for each topology measure employed by CePa

Name	Weight	In degree	Out degree	Betweenness	In reachability	Out reachability
(1) Pyrimidine biosynthesis	0.034	0.031	0.188	0.031	0.037	0.037
(2) Inactivation of APC C via direct inhibition of the APC C complex	0.031	0.047	0.047	0.037	0.037	0.037
(3) Removal of licensing factors from origins	0.031	0.034	0.031	0.037	0.037	0.037
(4) CDT1 association with the CDC6 ORC origin complex	0.031	0.0059	0.047	0.0058	0.037	0.037
(5) NCAM signaling for neurite out growth	0.031	0.034	0.098	0.083	0.037	0.037
(6) FGFR1c and Klotho ligand binding and activation	0.044	0.169	0.10	0.066	0.180	0.180
(7) Switching of origins to a post replicative state	0.031	0.034	0.031	0.037	0.037	0.037
(8) Fanconi Anemia pathway	0.031	0.034	0.031	0.037	0.037	0.037
(9) ChREBP activates metabolic gene expression	0.044	0.321	0.400	0.0058	0.338	0.338
(10) Telomere C strand Lagging Strand Synthesis	0.065	0.098	0.075	0.071	0.047	0.047

pathDIP in the colorectal cancer enrichment was able to identify 53 and 291 significant pathways (*p* value $< 0.005$) respectively from the *COAD* and *GSE41011* genes lists (let see Additional file 4 for the whole enriched pathway list). Table  4 shows the intersection of the first top 10 pathways (*p* value $< 0.005$) obtained from the two CC gene lists using the KEGG database.

**Table 4:** The intersection of the first 10 pathways (sorted by the relevance of *p* value $\le 0.005$) obtained from pathDIP by performing PEA using CC genes lists and the KEGG database

Pathway Name	*p* value	FDRc	Bc
(1) Pathways in cancer	1.93 × 10$^{-50}$	6.16 × 10$^{-48}$	6.16 × 10$^{-48}$
(2) Human papillomavirus infection	6.34 × 10$^{-32}$	1.01 × 10$^{-29}$	2.03 × 10$^{-29}$
(3) Endocytosis	4.28 × 10$^{-28}$	4.56 × 10$^{-26}$	1.37 × 10$^{-25}$
(4) Lysosome	8.40 × 10$^{-28}$	6.72 × 10$^{-26}$	2.69 × 10$^{-25}$
(5) MicroRNAs in cancer	8.88 × 10$^{-25}$	5.68 × 10$^{-23}$	2.84 × 10$^{-22}$
(6) Human T-cell leukemia virus 1 infection	1.42 × 10$^{-24}$	7.59 × 10$^{-23}$	4.56 × 10$^{-22}$
(7) MAPK signaling	1.94 × 10$^{-24}$	8.88 × 10$^{-23}$	6.22 × 10$^{-22}$
(8) PI3K-Akt signaling	5.31 × 10$^{-22}$	1.54 × 10$^{-20}$	1.70 × 10$^{-19}$
(9) Cell cycle	1.47 × 10$^{-21}$	3.92 × 10$^{-20}$	4.70 × 10$^{-19}$
(10) Autophagy-animal	1.92 × 10$^{-19}$	3.07 × 10$^{-18}$	6.13 × 10$^{-17}$

In the table FDRc represents the corrected *p* value using FDR corrector, and Bc refers to the corrected *p* value by using Bonferroni corrector

The pathDIP enrichment results include the following KEGG pathways. The *“Pathways in cancer”* is too general, making its role trivial in colorectal cancer. Fernandes et al. [58] assesses the role of *“Human papillomavirus infection”* in the onset and progression of colorectal cancer. Park et al. [68] describes the role of *“Endocytosis”* in colorectal cancer cells. Sun et al. [69] describes how the dichloroacetate attenuates the stemness of colorectal cancer cells via triggering ferroptosis through sequestering iron in *“lysosomes”* pathway. Grady et al. [70] describes a possible tumor suppressor role of *“microRNA”* in colorectal cancer due to the epigenetic silencing of the intronic microRNA hsa-miR-342 and its host gene EVL. We couldn’t find a link with the *“Human T-cell leukemia virus 1 infection”* and colorectal cancer. Slattery et al. [53] describes the involvement of *“MAPK signaling pathway”* in colorectal cancer. Agarwal et al. [71] assesses the role of *“PI3K-Akt signaling”* in cell survival and metastasis regulation in colorectal cancer. Mita et al. [72], Kuerbitz et al. [73] describe how *“Cell Cycle”* pathway is related to colorectal cancer. We did not investigate *“Autophagy - animal”* pathway because it is not related to the human species.

SPIA did not enrich any pathway from both CC genes lists (let see Additional file 5 for the whole enriched pathway list).

### Endometrial cancer enrichment using KEGG database

Analyzing with BiP the two datasets related to the Endometrial Cancer, that is *UCEC* and *GSE63678*, we have been able to identify 276 and 252 significant pathways (*p* value $\le 0.005$) respectively. Both enrichment results have been able to detect pathways linked to endometrial cancer (let see Additional file 2 for the whole enriched pathway list). Table  5 shows the BiP’s top 10 enriched pathways using both EC genes lists and the KEGG database.

The BiP enrichment results comprise the *“Metabolic”* pathway whose role in endometrial cancer is described in [74]. Liu et al. [75] shows the involvement of the *“Cytokine-cytokine receptor interaction”* in the development of endometrial cancer. The *Pathways in cancer* is too general, making its role obvious in endometrial cancer. Ouyang et al. [76] describes the role of *“Neuroactive ligand-receptor interaction”* with the endometrial cancer developing. Wu et al. [77] assesses the role of *“Transcriptional misregulation in cancer”* pathway in metastatic endometrial cancers development. Wang et al. [78] aimed to assess the potential role of long non-coding RNA BANCR promoting endometrial cancer cell proliferation and invasion by regulating MMP2 and MMP1 via ERK/*“MAPK signaling”* pathway. Yang et al. [79] describes how *“NOD-like receptors signaling pathway”* through Hydrogen could contribute to inhibit endometrial cancer growth. Kodati et al. [80] propose an hypothesis that *“shigellosis”* may be the trigger for the endometriosis. Winship et al. [81] describes the role of Chondroitin sulfate *“proteoglycan”* protein that stimulated by interleukin 11 promotes endometrial epithelial cancer cell proliferation and migration.

**Table 5:** The intersection of the first 10 pathways (sorted by the relevance of *p* value $\le 0.005$) obtained from BiP by performing PEA using EC genes lists and the KEGG database

Pathway Name	*p* value	FDRc	Bc
(1) Metabolic pathways	1.18 × 10$^{-199}$	3.21 × 10$^{-197}$	3.21 × 10$^{-197}$
(2) Cytokine-cytokine receptor interaction	3.14 × 10$^{-84}$	4.27 × 10$^{-82}$	8.53 × 10$^{-82}$
(3) Pathways in cancer	1.12 × 10$^{-49}$	1.01 × 10$^{-47}$	3.04 × 10$^{-47}$
(4) Neuroactive ligand-receptor interaction	5.60 × 10$^{-36}$	3.81 × 10$^{-34}$	1.52 × 10$^{-33}$
(5) Pathways of neurodegeneration-multiple diseases	2.09 × 10$^{-33}$	1.14 × 10$^{-31}$	5.69 × 10$^{-31}$
(6) Transcriptional misregulation in cancer	7.91 × 10$^{-33}$	3.59 × 10$^{-31}$	2.15 × 10$^{-30}$
(7) MAPK signaling pathway	4.41 × 10$^{-31}$	1.71 × 10$^{-29}$	1.20 × 10$^{-28}$
(8) NOD-like receptor signaling pathway	2.64 × 10$^{-28}$	8.98 × 10$^{-27}$	7.18 × 10$^{-26}$
(9) Shigellosis	9.48 × 10$^{-28}$	2.86 × 10$^{-26}$	2.58 × 10$^{-25}$
(10) Proteoglycans in cancer	1.94 × 10$^{-27}$	5.29 × 10$^{-26}$	5.29 × 10$^{-25}$

In the table, FDRc represents the corrected *p* value using FDR corrector, and Bc refers to the corrected *p* value using Bonferroni corrector

Table  6 shows the top 10 pathways obtained from CePa (let see Additional file 3 for the whole enriched pathway list). As shown, the first eight enriched pathways in Table  6 are identical to the ones obtained in colorectal cancer. Wang et al. [82] shows that reprogrammed branch chain amino-acid metabolism could promote endometrial cancer cells’ proliferation. While, for the *Removal of DNA patch containing an essential residue* pathway there are only few evidence of its involvement in the endometrial cancer [83, 84].

**Table 6:** The intersection of the first 10 pathways obtained from CePa by performing TEA using EC genes lists and the KEGG database. The table summarises the values obtained for each topology measure employed by CePa

Name	weight	in degree	out degree	betweenness	in reachability	out reachability
(1) Pyrimidine biosynthesis	0.031	0.029	0.146	0.037	0.0056	0.034
(2) Inactivation of APC C via direct inhibition of the APC C complex	0.044	0.0290	0.034	0.037	0.0056	0.034
(3) Removal of licensing factors from origins	0.031	0.029	0.034	0.037	0.047	0.034
(4) CDT1 association with the CDC6 ORC origin complex	0.031	0.029	0.066	0.062	0.047	0.034
(5) NCAM signaling for neurite out growth	0.031	0.029	0.034	0.085	0.121	0.034
(6) Switching of origins to a post replicative state	0.031	0.029	0.034	0.037	0.047	0.034
(7) Fanconi Anemia pathway	0.031	0.029	0.034	0.0377	0.047	0.041
(8) Telomere C strand Lagging Strand Synthesis	0.0056	0.062	0.066	0.0053	0.0056	0.041
(9) Branched chain amino acid catabolism	0.031	0.029	0.089	0.0053	0.047	0.034
(10) Removal of DNA patch containing a basic residue	0.031	0.062	0.034	0.085	0.087	0.034

Analyzing with pathDIP the two EC genes lists, that is, *UCEC* and *GSE63678*, we have been able to identify 133 and 60 significant pathways (*p* value $\le 0.005$) respectively. Both enrichment results have been able to detect pathways linked to endometrial cancer. Table  7 shows the pathDIP’s top 10 enriched pathways (*p* value $\le 0.005$) in both endometrial cancer genes lists (let see Additional file 4 for the whole enriched pathway list).

**Table 7:** The intersection of the first 10 pathways (sorted by the relevance of *p* value $\le 0.005$) obtained from pathDIP performing PEA using EC genes lists and the KEGG database

Pathway Name	*p* value	FDRc	Bc
(1) Alzheimer disease	2.47 × 10$^{-10}$	3.46 × 10$^{-08}$	6.93 × 10$^{-08}$
(2) Parkinson disease	7.46 × 10$^{-09}$	5.24 × 10$^{-07}$	2.10 × 10$^{-06}$
(3) Non-alcoholic fatty liver disease (NAFLD)	9.14 × 10$^{-08}$	5.13 × 10$^{-06}$	2.57 × 10$^{-05}$
(4) Cellular senescence	1.31 × 10$^{-06}$	4.60 × 10$^{-05}$	3.68 × 10$^{-04}$
(5) Huntington disease	4.98 × 10$^{-06}$	1.40 × 10$^{-04}$	1.40 × 10$^{-03}$
(6) Human T-cell leukemia virus 1 infection	8.80 × 10$^{-06}$	2.25 × 10$^{-04}$	2.47 × 10$^{-03}$
(7) Oxidative phosphorylation	9.73 × 10$^{-06}$	2.28 × 10$^{-04}$	2.73 × 10$^{-03}$
(8) Apoptosis	1.28 × 10$^{-05}$	2.76 × 10$^{-04}$	3.59 × 10$^{-03}$
(9) Mineral absorption	1.69 × 10$^{-05}$	3.17 × 10$^{-04}$	4.75 × 10$^{-03}$
(10) Cardiac muscle contraction	1.84 × 10$^{-05}$	3.24 × 10$^{-04}$	5.18 × 10$^{-03}$

In the table, FDRc represents the corrected *p* value using FDR corrector, and Bc refers to the corrected *p* value using Bonferroni corrector

Among the pathways enriched by pathDIP we could not find any link for the *“Alzheimer diseases”*, *“Parkinson diseases”*, *“Non-alcoholic fatty liver disease (NAFLD)”*, *“Human T-cell leukemia virus 1 infection”*, *“Cardiac muscle contraction”* pathways and the endometrial cancer. Konno et al. [85] delineates the role of *“Cellular senescence”* pathway in suppressing proliferating, and stem cell-like phenotype of aggressive endometrial cancer cells. Nevadunsky et al. [86] assesses the effects of such *“oxidative phosphorylation”* in both endometrial and non-endometrial cancer types. Wong et al. [87] demonstrate that treatment based on Dichloroacetate promotes *“apoptosis”* in endometrial cancer. Huo et al. [88] describes the involvement of *“Mineral absorption”* pathway in the underlying biological mechanisms driving the tumorgenesis of endometrial cancer.

SPIA did not enrich any pathway from both EC genes lists (let see Additional file 5 for the whole enriched pathway list).

### Thyroid cancer enrichment using KEGG database

Analyzing with BiP the two thyroid cancer genes lists, that is, *THCA* and *GSE65144*, we have been able to identify 280 and 54 significant pathways (*p* value $< 0.005$) respectively. Both BiP’s enrichment results have been able to detect thyroid cancer-associated pathways. Table  8 shows the BiP’s top 10 overlapping pathways (*p* value $\le 0.005$) obtained from the two TC genes lists and the KEGG database (let see Additional file 2 for the whole enriched pathway list).

Searching the literature, we found the following evidence for the enriched pathways listed in Table  8. Filetti et al. [89] shows that the intrathyroidal iodine *“metabolism pathway”* represents one of the most peculiar abnormalities present in neoplastic thyroid tissue. Feng et al. [90] describes the role of *“Cytokine-cytokine receptor interaction”* with thyroid cancer. The *“Pathways in cancer”* is too general, making its role obvious in thyroid cancer. To the best of our knowledge, we could not find any link between *“Pathways of neurodegeneration - multiple diseases”*, *“Human immunodeficiency virus 1 infection”* and thyroid cancer. Han et al. [91] delineates the link between *“Ubiquitin mediated proteolysis”* pathway and thyroid cancer. Xu et al. [92] describes the involvement of *“neuroactive ligand-receptor interaction”* in thyroid cancer. Bonara et al. [93] describe the defective *“oxidative phosphorylation”* in thyroid cancer associated with pathogenic mitochondrial DNA mutations. [94] describes the link between $LXR\beta$ and *ribosome* activity to develop new diagnostic and therapeutic targets in thyroid cancers. Zhao et al. [95] presents how the tunicamycin promotes metastasis through up-regulating *“endoplasmic reticulum”* in thyroid carcinoma.

**Table 8:** The intersection of the first 10 pathways (sorted by the relevance of *p* value $\le 0.005$) obtained from BiP, performing PEA using TC genes lists and the KEGG database

Pathway Name	*p* value	FDRc	Bc
(1) Metabolic pathways	1.59 × 10$^{-111}$	4.98 × 10$^{-109}$	4.98 × 10$^{-109}$
(2) Cytokine-cytokine receptor interaction	1.02 × 10$^{-40}$	1.60 × 10$^{-38}$	3.21 × 10$^{-38}$
(3) Pathways in cancer	2.50 × 10$^{-30}$	2.62 × 10$^{-28}$	7.85 × 10$^{-28}$
(4) Pathways of neurodegeneration-multiple diseases	7.56 × 10$^{-26}$	5.93 × 10$^{-24}$	2.37 × 10$^{-23}$
(5) Ubiquitin mediated proteolysis	1.75 × 10$^{-17}$	3.44 × 10$^{-16}$	5.50 × 10$^{-15}$
(6) Neuroactive ligand-receptor interaction	2.72 × 10$^{-17}$	4.75 × 10$^{-16}$	8.56 × 10$^{-15}$
(7) Oxidative phosphorylation	7.94 × 10$^{-17}$	1.25 × 10$^{-15}$	2.49 × 10$^{-14}$
(8) Ribosome	2.02 × 10$^{-16}$	2.89 × 10$^{-15}$	6.35 × 10$^{-14}$
(9) Protein processing in endoplasmic reticulum	1.39 × 10$^{-15}$	1.62 × 10$^{-14}$	4.37 × 10$^{-13}$
(10) Human immunodeficiency virus 1 infection	2.06 × 10$^{-15}$	2.16 × 10$^{-14}$	6.47 × 10$^{-13}$

In the table, *FDRc* represents the corrected *p* value using FDR corrector, and *Bc* refers to the corrected *p* value using Bonferroni corrector

CePa enrichment shows nine pathways in common between colorectal and endometrial cancer. The number of shared genes among the six analyzed cancer data sets is equal to 30, a value that is not enough to support this overlap in the pathways enrichment results, obtained using these different genes lists. Table  9 shows the CePa’s top 10 pathways obtained from the intersection of the pathway enrichment results for the two TC genes lists and the KEGG database (let see Additional file 3 for the whole enriched pathway list). The remaining, *“Generation of second messenger molecules”* in [96] describes as the *PIP3* acts as the second messenger into the thyroid cancer.

Analyzing with pathDIP the two thyroid cancer genes lists, that is, *THCA* and *GSE65144*, we have been able to identifying 71 and 223 significant pathways (*p* value $\le 0.005$) respectively (let see Additional file 4 for the whole enriched pathway list). Both pathDIP’s enrichment results, have been able to detect thyroid cancer associated pathways. Table  10 shows the pathDIP’s top 10 overlapping pathways (*p* value $< 0.005$) from the two TC genes lists and the KEGG database.

**Table 9:** The 10 enriched pathways with CePa using the two TC genes lists and the KEGG database. The table summarises the values obtained for each topology measure employed by CePa

Name	Weight	In degree	Out degree	Betweenness	In reachability	Out reachability
(1) Pyrimidine biosynthesis	0.034	0.034	0.180	0.041	0.034	0.029
(2) Inactivation of APC C via direct inhibition of the APC C complex	0.047	0.0050	0.029	0.041	0.034	0.047
(3) Generation of second messenger molecules	0.034	0.097	0.0059	0.118	0.091	0.118
(4) Removal of licensing factors from origins	0.034	0.034	0.029	0.041	0.034	0.029
(5) CDT1 association with the CDC6 ORC origin complex	0.047	0.034	0.041	0.0058	0.071	0.029
(6) Association of licensing factors with the pre replicative complex	0.205	0.034	0.188	1	0.066	0.233
(7) NCAM signaling for neurite out growth	0.034	0.062	0.029	0.068	0.091	0.029
(8) Switching of origins to a post replicative state	0.034	0.034	0.029	0.041	0.034	0.029
(9) Fanconi Anemia pathway	0.034	0.034	0.029	0.041	0.034	0.029
(10) Telomere C strand Lagging Strand Synthesis	0.0051	0.065	0.041	0.082	0.075	0.029

**Table 10:** The intersection of the first 10 pathways (sorted by the relevance of *p* value $\le 0.005$) obtained from pathDIP performing PEA using TC genes lists and the KEGG database

Pathway Name	*p* value	FDRc	Bc
(1) MicroRNAs in cancer	1.95 × 10$^{-29}$	6.18 × 10$^{-27}$	6.18 × 10$^{-27}$
(2)Endocytosis	2.82 × 10$^{-27}$	4.47 × 10$^{-25}$	8.94 × 10$^{-25}$
(3)Pathways in cancer	2.71 × 10$^{-25}$	2.86 × 10$^{-23}$	8.59 × 10$^{-23}$
(4)PI3K-Akt signaling	4.67 × 10$^{-24}$	3.70 × 10$^{-22}$	1.48 × 10$^{-21}$
(5)Proteoglycans in cancer	1.04 × 10$^{-21}$	6.58 × 10$^{-20}$	3.29 × 10$^{-19}$
(6)Human papillomavirus infection	5.76 × 10$^{-19}$	2.61 × 10$^{-17}$	1.83 × 10$^{-16}$
(7)Focal adhesion	5.79 × 10$^{-17}$	2.29 × 10$^{-15}$	1.84 × 10$^{-14}$
(8)MAPK signaling	7.28 × 10$^{-16}$	2.31 × 10$^{-14}$	2.31 × 10$^{-13}$
(9)Phagosome	2.29 × 10$^{-15}$	6.59 × 10$^{-14}$	7.25 × 10$^{-13}$
(10)Regulation of actin cytoskeleton	8.49 × 10$^{-15}$	1.92 × 10$^{-13}$	2.69 × 10$^{-12}$

In the table, *FDRc* represents the corrected *p* value using FDR corrector, and *Bc* refers to the corrected *p* value using Bonferroni corrector

To the best of our knowledge we could not find any study directly linking *“MicroRNAs in cancer”*, *“Proteoglycans in cancer”*, *“Human papillomavirus infection”*, *“phagosome”*, *“Regulation of actin cytoskeleton”* and the thyroid cancer. The *“Pathways in cancer”* is too general, making its role obvious in thyroid cancer. Theret et al. [97] describes the identification of LRP-1 as an *“endocytosis”* and recycling receptor for $\beta$1-integrin in thyroid cancer cells. Liu et al. [98] describes how the LncRNA modulates the cell proliferation and cancer growth of thyroid cancer through *“PI3K-Akt signaling”* pathway. Owens et al. [99] explained the possible role of *“Focal adhesion”* in a mechanism for metastasis of thyroid cancer. Eissing et al. [100] describes how Notch pathway activation by the *“MAPK signaling”* is responsible for thyroid cancer proliferation.

SPIA did not enrich any pathway from both TC genes lists (let see Additional file 5 for the whole enriched pathway list).

### Colorectal cancer enrichment using Reactome database

Analyzing with BiP the two genes lists related to colorectal cancer -*COAD* and *GSE41011* - and the Reactome database we identified 586 and 896 significant pathways (*p* value $< 0.005$) respectively. The enrichment results obtained by BiP point out its capability to identify some well-known colon cancer risk pathways. Table  11 shows the top 10 pathways (*p* value $< 0.005$) obtained by the intersection results from the two CC genes lists and the Reactome database.

**Table 11:** The intersection of the first 10 pathways (sorted by the relevance of *p* value $\le 0.005$) obtained from BiP performing PEA using CC genes lists and the Reactome database

Pathway Name	*p* value	FDRc	Bc
(1) Metabolism of proteins	2.32 × 10$^{-63}$	4.66 × 10$^{-60}$	4.66 × 10$^{-60}$
(2) Post-translational protein modification	2.13 × 10$^{-49}$	2.14 × 10$^{-46}$	4.29 × 10$^{-46}$
(3) Cellular responses to external stimuli	5.70 × 10$^{-35}$	3.82 × 10$^{-32}$	1.14 × 10$^{-31}$
(4) Cell Cycle, Mitotic	1.90 × 10$^{-33}$	9.57 × 10$^{-31}$	3.83 × 10$^{-30}$
(5) Mitotic G1-G1/S phases	1.90 × 10$^{-33}$	7.65 × 10$^{-31}$	3.83 × 10$^{-30}$
(6) Cellular responses to stress	1.98 × 10$^{-33}$	6.63 × 10$^{-31}$	3.98 × 10$^{-30}$
(7) Cell Cycle	3.03 × 10$^{-33}$	8.71 × 10$^{-31}$	6.10 × 10$^{-30}$
(8) Mitotic G2-G2/M phases	9.07 × 10$^{-32}$	2.28 × 10$^{-29}$	1.82 × 10$^{-28}$
(9) DNA Repair	6.35 × 10$^{-31}$	1.42 × 10$^{-28}$	1.28 × 10$^{-27}$
(10) Cell Cycle Checkpoints	6.55 × 10$^{-31}$	1.32 × 10$^{-28}$	1.32 × 10$^{-27}$

In the table, *FDRc* represents the corrected *p* value using FDR corrector, and *Bc* refers to the corrected *p* value using Bonferroni corrector

Hughes et al. [101] describes the associations between *“Metabolism of Proteins”* pathway and the colorectal cancer. Jaén et al. [102], Tomonaga et al. [103] describe the role of *“The Post-translational protein modification pathway”* in the colorectal cancer. Fang et al. [104] describes the *“Cellular responses to external stimuli”* as a general pathway that regulates how a single cell detects and responds to external molecular and physical signals, comprising the mitogen-activated protein kinases (MAPK), the extracellular-signal-regulated kinases in colorectal cancer. Mita et al. [72], Kuerbitz et al. [73] desribe how the *“Cell Cycle, Mitotic”*, *“Cell Cycle”* and *“Cell Cycle Checkpoints”* pathways, are related to colorectal cancer. Furthermore, *survivin-transcription* is controlled by specific sequences in the promoter region, and it increases during *“Mitotic G1-G1/S phases”* [72, 105], and reaches a peak in *“Mitotic G2-G2/M phases”* [72, 106], other two pathways enriched by BiP placed in the first 10 positions by *p* value relevance. A recent manuscript published by Reilly et al. [107] describes as the alterations in DNA repair genes could provide new therapeutic opportunities for colorectal cancer, that is a further evidence for the *“DNA repair”* pathway in colorectal cancer enriched by BiP. Finally, [108] describes the role of *“Cellular responses to stress”* pathway in colorectal cancer.

Analyzing with pathDIP the two genes lists related to colorectal cancer, that is, *COAD* and *GSE65144*, we identified 59 and 704 significant pathways (*p* value $\le 0.005$) respectively. Both pathDIP’s enrichment results, have been able to detect colorectal cancer associated pathways. Table  12 shows the top 10 overlapping pathways (*p* value $\le 0.005$) obtained from the two enrichment analysis using Reactome database.

**Table 12:** The intersection of the first 10 pathways (sorted by the relevance of *p* value $\le 0.005$) obtained from pathDIP performing PEA using CC genes lists and the Reactome database

Pathway Name	*p* value	FDRc	Bc
(1) Metabolism of proteins	3.18 × 10$^{-74}$	6.31 × 10$^{-71}$	6.31 × 10$^{-71}$
(2) Immune System	1.46 × 10$^{-60}$	9.64 × 10$^{-58}$	2.89 × 10$^{-57}$
(3) Post-translational protein modification	6.40 × 10$^{-49}$	3.17 × 10$^{-46}$	1.27 × 10$^{-45}$
(4) Disease	4.02 × 10$^{-37}$	1.33 × 10$^{-34}$	7.96 × 10$^{-34}$
(5) Gene expression (Transcription)	1.34 × 10$^{-36}$	3.79 × 10$^{-34}$	2.66 × 10$^{-33}$
(6) RNA Polymerase II Transcription	1.01 × 10$^{-29}$	2.00 × 10$^{-27}$	2.00 × 10$^{-26}$
(7) Innate Immune System	4.51 × 10$^{-27}$	8.13 × 10$^{-25}$	8.95 × 10$^{-24}$
(8) Cell Cycle	2.07 × 10$^{-25}$	2.93 × 10$^{-23}$	4.11 × 10$^{-22}$
(9) Generic Transcription Pathway	2.94 × 10$^{-25}$	3.88 × 10$^{-23}$	5.83 × 10$^{-22}$
(10) Transcriptional Regulation by TP53	3.27 × 10$^{-21}$	3.09 × 10$^{-19}$	6.48 × 10$^{-18}$

In the table, *FDRc* represents the corrected *p* value using FDR corrector, and *Bc* refers to the corrected *p* value using Bonferroni corrector

Hughes et al. [101] delineates the associations between *“Metabolism of Proteins”* pathway and the colorectal cancer. [109] shows how the *“immune system”* pathway plays an integral role in preventing and promoting the development of colorectal cancer. Jaén et al. [102] and Tomonaga et al. [103] show the role of *“The Post-translational protein modification pathway”* in colorectal cancer. At the best of our knowledge, it was not possible to identify any link between *“disease”*, *“Generic Transcription Pathway”*, *“Transcriptional Regulation by TP53”* pathways and colorectal cancer. [110] investigates the effect of PI3K pathway in regulating colorectal cancer cell lines and the *“gene expression”* pathway. Wang et al. [111] shows that *“RNA polymerase II transcription”* through BTF3 contributes to primary colorectal cancer or metastasis. [112] discusses the mechanisms of colitis and colitis-associated colorectal cancer used by the innate immune system in the intestine. Mita et al. [72], Kuerbitz et al. [73] describes how the *“Cell Cycle”* pathway is related to colorectal cancer.

### Endometrial cancer enrichment using Reactome database

Analyzing with BiP the two genes lists related to the Endometrial Cancer , that is, *UCEC* and *GSE63678*, we have been able to identify 566 and 1, 173 significant pathways (*p* value $\le 0.005$) respectively. Both enrichment results have been able to detect pathways linked to endometrial cancer. Table  13 shows the top 10 enriched pathways (*p* value $\le 0.005$) obtained using both EC genes lists and the Reactome database.

**Table 13:** The intersection of the first 10 pathways (sorted by the relevance of *p* value $\le 0.005$) obtained from BiP performing PEA using EC genes lists and the Reactome database

Pathway Name	*p* value	FDRc	Bc
(1) Metabolism of proteins	2.35 × 10$^{-182}$	4.83 × 10$^{-179}$	4.83 × 10$^{-179}$
(2) Metabolism	3.89 × 10$^{-173}$	4.00 × 10$^{-170}$	8.00 × 10$^{-170}$
(3) Cellular responses to external stimuli	5.22 × 10$^{-160}$	3.57 × 10$^{-157}$	1.07 × 10$^{-156}$
(4) Signaling by Receptor Tyrosine Kinases	2.82 × 10$^{-159}$	1.45 × 10$^{-156}$	5.80 × 10$^{-156}$
(5) Cellular responses to stress	5.82 × 10$^{-157}$	2.39 × 10$^{-154}$	1.20 × 10$^{-153}$
(6) Cell Cycle	1.30 × 10$^{-151}$	4.46 × 10$^{-149}$	2.67 × 10$^{-148}$
(7) Post-translational protein modification	3.30 × 10$^{-150}$	9.68 × 10$^{-148}$	6.77 × 10$^{-147}$
(8) Cell Cycle, Mitotic	7.04 × 10$^{-150}$	1.81 × 10$^{-147}$	1.44 × 10$^{-146}$
(9) Mitotic G1-G1/S phases	7.04 × 10$^{-150}$	1.61 × 10$^{-147}$	1.44 × 10$^{-146}$
(10) Cellular Senescence	3.54 × 10$^{-149}$	7.27 × 10$^{-147}$	7.27 × 10$^{-146}$

In the table, *FDRc* represents the corrected *p* value using FDR corrector, and *Bc* refers to the corrected *p* value using Bonferroni corrector

Mehlen et al. [113] describes the connections between *“Axon guidance”* and endometrial cancer. Baylin et al. [114], Mäkinen et al. [115] report the relationships between transcription pathways (*“Gene expression (Transcription)”*, *“RNA Polymerase II Transcription”* and *“Generic Transcription Pathway”*) with endometrial cancer. Takai et al. [116] shows the connection between *“Signaling by Receptor Tyrosine Kinases”* and endometrial cancer. Yu et al. [117], Change et al. [118, 119] prove the link between *“ERBB2 and ERBB4 signaling pathways”* and the endometrial cancer, while [120] reviews the role of *“Rho GTPases signaling”* in cancer. Deregulation of the cell cycle is famously linked to cancer development [121], while the bi-directional correlation between the neural factors and cancer progression and metastasis is more recent [122].

Analyzing with pathDIP the two genes lists related to the Endometrial Cancer, that is, *UCEC* and *GSE63678*, we identified 22 and 215 significant pathways (*p* value $\le 0.005$) respectively. The pathway enrichment intersection obtained from pathDIP using the two EC genes lists and the Reactome database, contains only 7 pathways in common. Table  14 shows the top 7 enriched pathways (*p* value $< 0.005$) obtained using EC genes lists and Reactome database.

**Table 14:** The intersection of the first 7 pathways (sorted by the relevance of *p* value $\le 0.005$) obtained from pathDIP performing PEA using EC genes lists and the Reactome database

Pathway Name	*p* value	FDRc	Bc
(1) RNA Polymerase II Transcription	5.04 × 10$^{-11}$	7.43 × 10$^{-09}$	5.94 × 10$^{-08}$
(2) Gene expression (Transcription)	6.83 × 10$^{-11}$	8.95 × 10$^{-09}$	8.06 × 10$^{-08}$
(3) Generic Transcription Pathway	1.67 × 10$^{-10}$	1.51 × 10$^{-08}$	1.97 × 10$^{-07}$
(4) Metabolism of proteins	6.64 × 10$^{-07}$	2.70 × 10$^{-05}$	7.83 × 10$^{-04}$
(5) Immune System	6.85 × 10$^{-06}$	2.07 × 10$^{-04}$	8.09 × 10$^{-03}$
(6) Innate Immune System	4.79 × 10$^{-03}$	3.25 × 10$^{-02}$	1.00$\times 10^{+00}$
(7) Translation	7.01 × 10$^{-03}$	4.31 × 10$^{-02}$	1.00$\times 10^{+00}$

In the table, *FDRc* represents the corrected *p* value using FDR corrector, and *Bc* refers to the corrected *p* value using Bonferroni corrector

Baylin et al. [114], and Mäkinen et al. [115] delineate the relationships between transcription pathways (*“Gene expression Transcription”*, *“RNA Polymerase II Transcription”* and *“Generic Transcription Pathway”*) with endometrial cancer. At the best of our knowledge, it was not possible to identify any link between *“Metabolism of proteins”*, *“Innate Immune System”*, and *“Translation”* pathways with endometrial cancer. [123] delineates the implications of *“immune system”* in endometrial cancer development.

### Thyroid cancer enrichment using Reactome database

Analyzing with BiP the two thyroid cancer genes lists that is, *THCA* and *GSE65144*, we identified 374 and 921 relevant pathways (*p* value $\le 0.005$) respectively. Both enrichment results, have been able to detect thyroid cancer associated pathways. Table  15 shows the top 10 overlapping pathways obtained enriching the two TC genes lists and using Reactome database.

**Table 15:** The first 10 enriched pathways (sorted by the relevance of *p* value $\le 0.005$) obtained from BiP performing PEA using TC genes lists and the Reactome database

Pathway Name	*p* value	FDRc	Bc
(1) Mitotic Prometaphase	7.74 × 10$^{-14}$	2.52 × 10$^{-11}$	1.51 × 10$^{-10}$
(2) Membrane Trafficking	3.61 × 10$^{-12}$	8.80 × 10$^{-10}$	7.04 × 10$^{-09}$
(3) Signaling by Rho GTPases	7.67 × 10$^{-12}$	1.15 × 10$^{-09}$	1.50 × 10$^{-08}$
(4) Post-translational protein modification	1.49 × 10$^{-11}$	1.94 × 10$^{-09}$	2.91 × 10$^{-08}$
(5) Extracellular matrix organization	1.40 × 10$^{-10}$	1.36 × 10$^{-08}$	2.72 × 10$^{-07}$
(6) Metabolism of proteins	2.48 × 10$^{-10}$	2.11 × 10$^{-08}$	4.85 × 10$^{-07}$
(7) M Phase	3.18 × 10$^{-10}$	2.48 × 10$^{-08}$	6.20 × 10$^{-07}$
(8) Cell Cycle, Mitotic	1.17 × 10$^{-09}$	7.86 × 10$^{-08}$	2.28 × 10$^{-06}$
(9) Mitotic G1-G1/S phases	1.17 × 10$^{-09}$	7.59 × 10$^{-08}$	2.28 × 10$^{-06}$
(10) Cell Cycle	1.58 × 10$^{-09}$	9.64 × 10$^{-08}$	3.08 × 10$^{-06}$

In the table, *FDRc* represents the corrected *p* value using FDR corrector, and *Bc* refers to the corrected *p* value using Bonferroni corrector

Nucera et al. [124] reports the link between *“Extracellular matrix organization”* and thyroid cancer. Zhong et al. [125] describes the involvement of *“RHO GTPases”* in thyroid cancer, while [126] describes the connection between *“Membrane Trafficking”* pathway and thyroid cancer. Interestingly, few pathways are linked to cell cycle mentioned above, that is well known to be deregulated in cancer (pathways: *“Mitotic Prometaphase”*, *“M Phase”*, *“Cell Cycle, Mitotic”*, *“Mitotic G1-G1/S phase”*, *“Cell Cycle”*). Ząbczyńska et al. [127] describes the link between Changes in the glycosylation profile (a popular type of post-translational modification (PTM) ) pathway with thyroid cancer. While we couldn’t find a link with the metabolism of proteins pathway.

Analyzing with pathDIP the two genes lists related to thyroid cancer, that is, *THCA* and *GSE65144*, we identified 28 and 405 relevant pathways (*p* value $\le 0.005$) respectively. Both enrichment results, have been able to detect thyroid cancer associated pathways. Table  16 shows the top 10 overlapping pathways (*p* value $\le 0.005$) using the two TC genes lists and Reactome database.

**Table 16:** The first 10 enriched pathways (sorted by the relevance of *p* value $\le 0.005$) obtained from pathDIP performing PEA using TC genes lists and the Reactome database

Pathway Name	*p* value	FDRc	Bc
(1) Immune System	1.56 × 10$^{-28}$	2.90 × 10$^{-25}$	2.90 × 10$^{-25}$
(2) Metabolism	9.59 × 10$^{-28}$	5.94 × 10$^{-25}$	1.78 × 10$^{-24}$
(3) Metabolism of proteins	6.89 × 10$^{-27}$	3.20 × 10$^{-24}$	1.28 × 10$^{-23}$
(4) Cell Cycle, Mitotic	5.72 × 10$^{-26}$	1.52 × 10$^{-23}$	1.06 × 10$^{-22}$
(5) Post-translational protein modification	3.01 × 10$^{-24}$	6.20 × 10$^{-22}$	5.58 × 10$^{-21}$
(6) Membrane Trafficking	1.37 × 10$^{-21}$	2.54 × 10$^{-19}$	2.54 × 10$^{-18}$
(7) Neutrophil degranulation	3.17 × 10$^{-18}$	4.91 × 10$^{-16}$	5.90 × 10$^{-15}$
(8) M Phase	5.68 × 10$^{-16}$	4.59 × 10$^{-14}$	1.06 × 10$^{-12}$
(9) Disease	7.65 × 10$^{-14}$	5.07 × 10$^{-12}$	1.42 × 10$^{-10}$
(10) Signaling by Receptor Tyrosine Kinases	4.96 × 10$^{-13}$	2.88 × 10$^{-11}$	9.21 × 10$^{-10}$

In the table, *FDRc* represents the corrected *p* value using FDR corrector, and *Bc* refers to the corrected *p* value using Bonferroni corrector

[126] describes the connection between *“Membrane Trafficking”* pathway and thyroid cancer. Interestingly, few pathways are linked to *“cell cycle”* mentioned above, that is well known to be deregulated in cancer (pathways: *“Mitotic Prometaphase”*, *“M Phase”*, *“Cell Cycle, Mitotic”*, *“Mitotic G1-G1/S phase”*, *“Cell Cycle”*). We couldn’t find a link between the *“Metabolism”*, *“metabolism of proteins”*, *“Immune System”*, *“Post-translational protein modification”*, *“Neutrophil degranulation”*, *“Disease”* pathways and thyroid cancer.

### Pathway enrichment similarity assessment

To compare the pathway enrichment results obtained from BiP with CePa, pathDIP, and SPIA, we used the same six gene lists obtained from the cancer datasets listed in Table 1, along with the KEGG and Reactome databases.

For each couple of enrichments, we measured the total number of the enriched pathway with *p* value $\le 0.005$, and the number of enriched pathways that belong to the union, and the intersection between the enriched results, concerning the used database. These information have been summarized in Tables 17 and 18.

**Table 17:** The number of enriched pathways obtained by using the six genes datasets and the KEGG database

Tool	CC				EC				TC			
	TCGA	GEO			TCGA	GEO			TCGA	GEO		
	NP	NP	U	I	NP	NP	U	I	NP	NP	U	I
BiP	274	53	274	53	276	252	295	233	280	54	284	50
CePa	23	25	27	21	23	25	27	21	23	25	28	20
pathDIP	52	290	291	51	133	60	152	41	71	233	226	68
SPIA	4	1	5	0	1	6	7	0	0	19	19	0

NP stands for Number of Enriched Pathways, U is short for Union, and I is short for Intersection

**Table 18:** The number of enriched pathways obtained by using the six genes datasets and the Reactome database

Tool	CC				EC				TC			
	TCGA	GEO			TCGA	GEO			TCGA	GEO		
	NP	NP	U	I	NP	NP	U	I	NP	NP	U	I
BiP	586	896	936	546	566	1173	1184	555	374	921	930	365
pathDIP	59	703	730	32	22	215	230	7	28	405	414	19

NP stands for Number of Enriched Pathways, U is short for Union, and I is short for Intersection

**Fig. 1:**
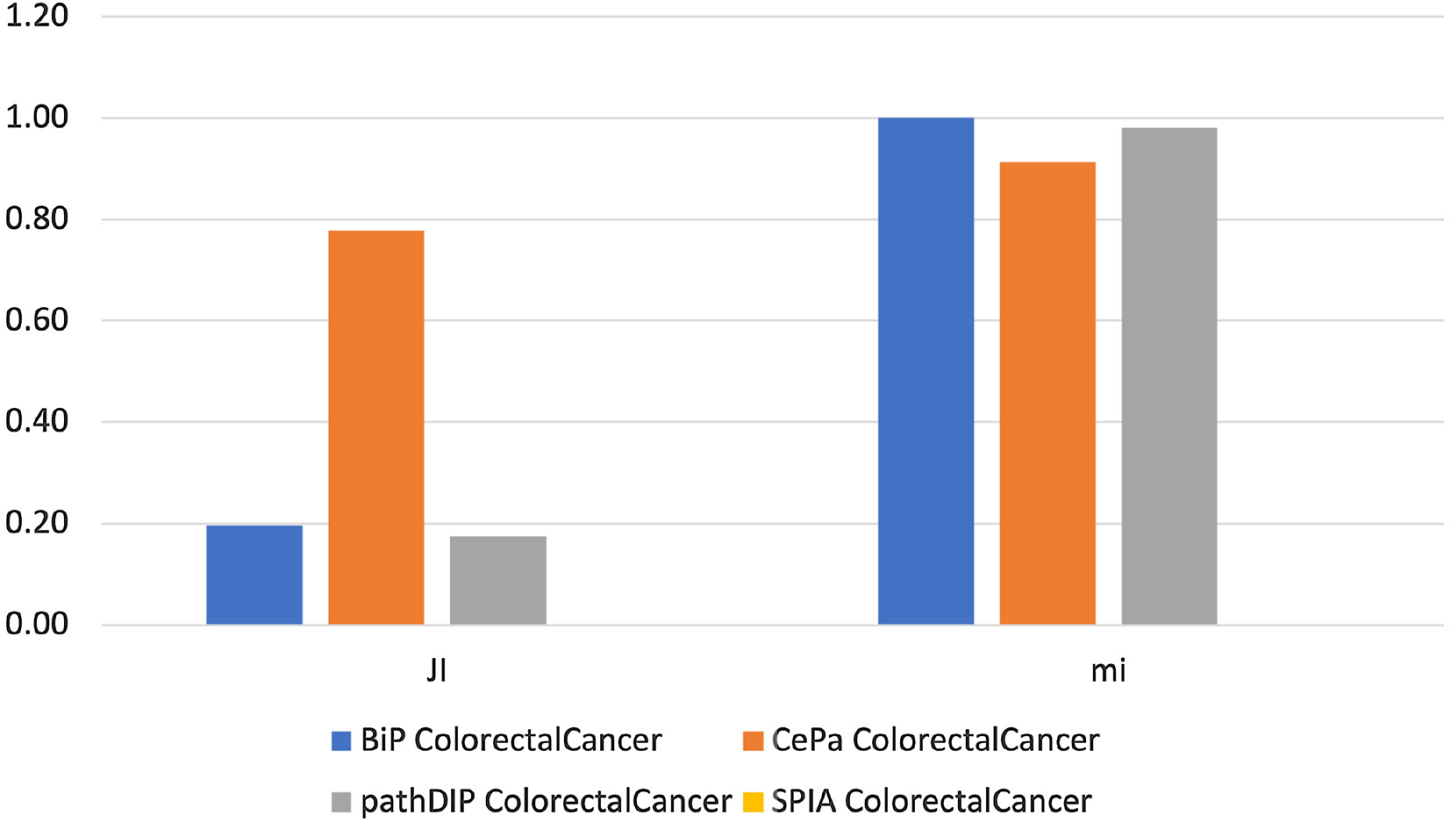
The similarity score obtained by each tool analyzing both CC gene lists using the KEGG database. The histograms show the score reached by each tool in percentage. In the Figure, JI indicates the Jaccard Index, which measures the similarity of two classes of samples. mi corresponds to the meet-min index used to quantify the containment between two sets

**Fig. 2:**
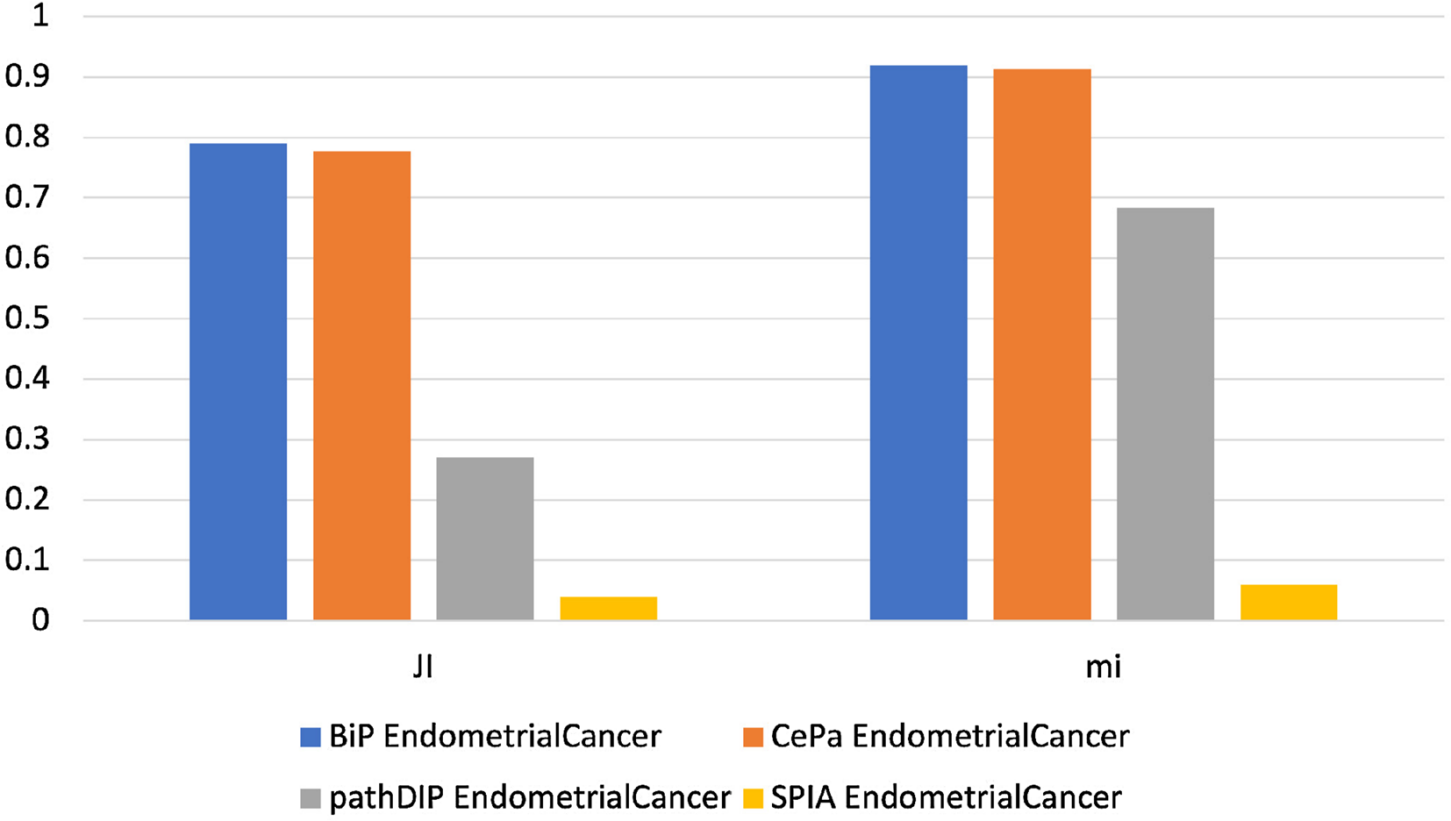
The similarity score obtained by each tool analyzing both EC gene lists using the KEGG database. The histograms show the score reached by each tool in percentage. In the Figure, JI indicates the Jaccard Index, which measures the similarity of two classes of samples. mi corresponds to the meet-min index used to quantify the containment between two sets

**Fig. 3:**
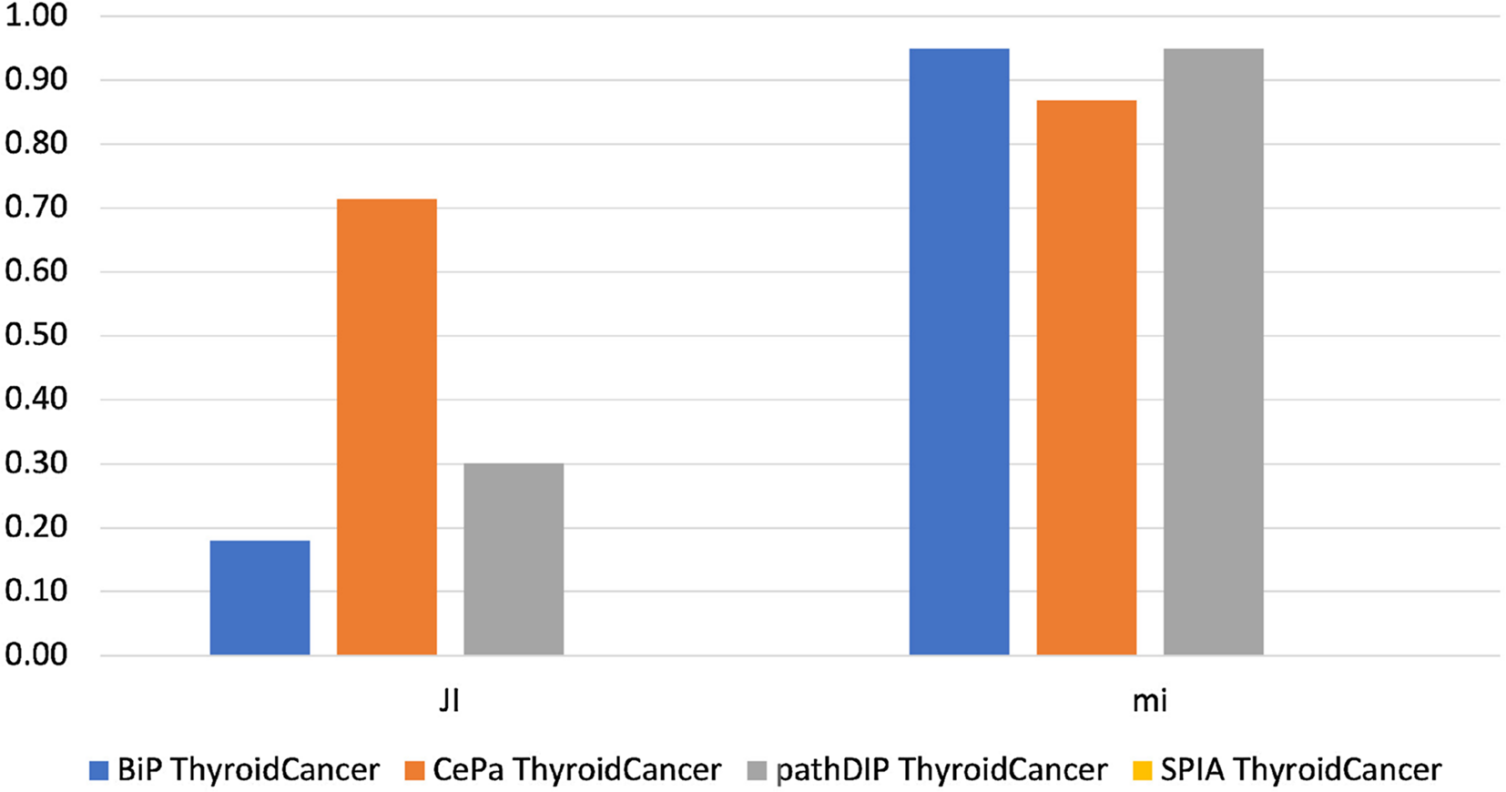
The similarity score, obtained by each tool analyzing both TC gene lists using the KEGG database. The histograms show the score reached by each tool in percentage. In the Figure, JI indicates the Jaccard Index, which measures the similarity of two classes of samples. mi corresponds to the meet-min index used to quantify the containment between two sets

**Fig. 4:**
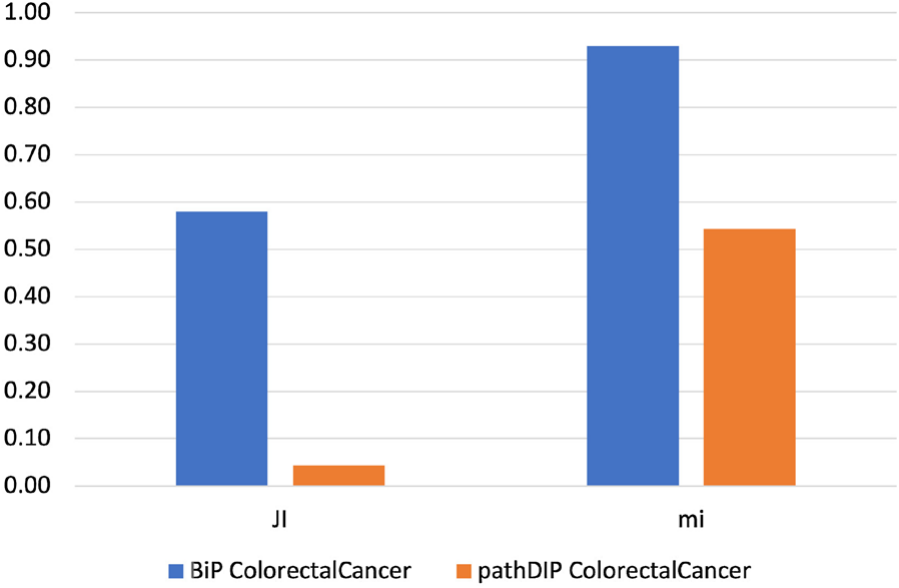
The similarity score obtained by each tool analyzing both CC gene lists using the Reactome database. The histograms show the score reached by each tool in percentage. In the Figure, JI indicates the Jaccard Index, which measures the similarity of two classes of samples. mi corresponds to the meet-min index used to quantify the containment between two sets

**Fig. 5:**
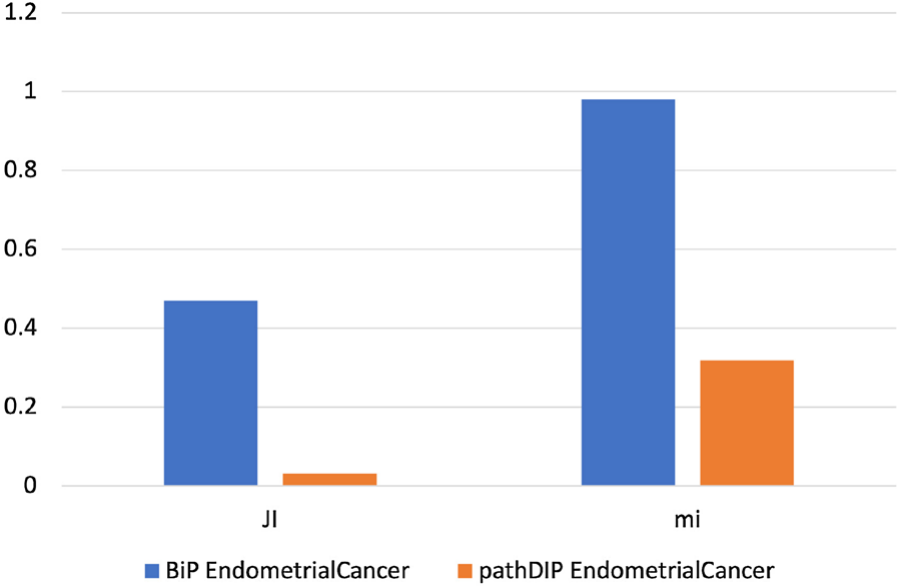
The similarity score obtained by each tool analyzing both EC gene lists using the Reactome database. The histograms show the score reached by each tool in percentage. In the Figure, JI indicates the Jaccard Index, which measures the similarity of two classes of samples. mi corresponds to the meet-min index used to quantify the containment between two sets

**Fig. 6:**
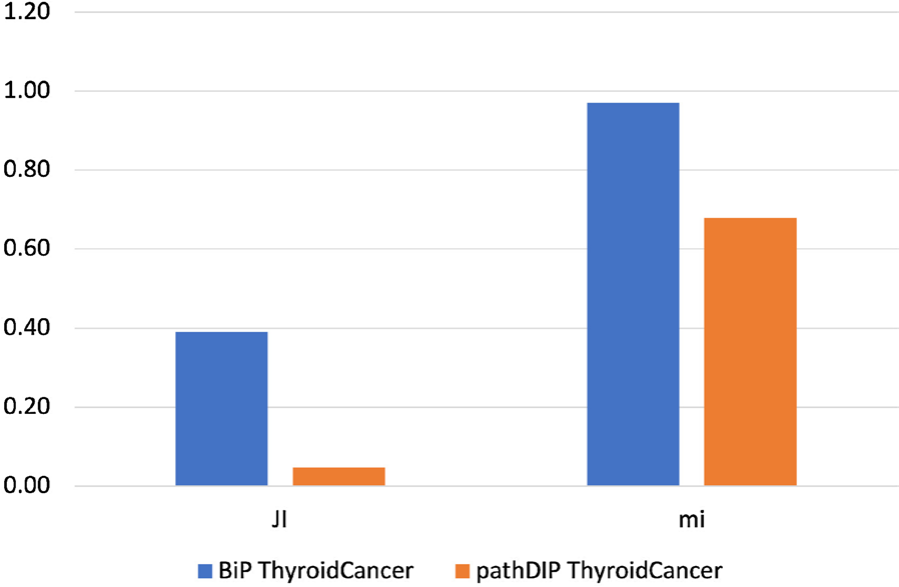
The similarity score obtained by each tool analyzing both TC gene lists using the Reactome database. The histograms show the score reached by each tool in percentage. In the Figure, JI indicates the Jaccard Index, which measures the similarity of two classes of samples. mi corresponds to the meet-min index used to quantify the containment between two set

To measure the similarity between the pairs of pathway enrichments obtained from the investigated cancer genes lists, we adopted the *Jaccard* index able to measure the similarity between two sets and the *meet-min* index that can assess the containment property between two groups. High *Jaccard* index values indicate that the two enrichments are similar, while high values of *meet-min* index suggest that the smallest set is contained in the biggest. Figures  1, 2, 3, 4, 5 and 6 show the *Jaccard* and *meet-min* indices values respectively for each software tool applied using KEGG or Reactome, to each cancer data set.

Results show that BiP achieves the highest *Jaccard* and *meet-min* indexes values in almost all comparisons, revealing that BiP is more stable than the other methods. Only in two cases, CePa obtains better *Jaccard* values when enriching CC and TC gene lists. Result due to the relatively similar number of enriched pathways in the two enrichment results (see Table  17), indicating that the two sets are only in the overlap. Conversely, BiP obtains higher values of *meet-min*, a most suitable measure of containment between two groups.

The better enrichment stability yielded by BiP may be due to the use of updated pathway information. Pathway enrichment analysis is influenced by outdated pathway information, and the used database [26]. Outdated pathway information negatively affects pathway enrichment analysis. To use updated pathway information, BiP allows users to load and use the last version of pathway data available from Reactome, KEGG, and the other web databases compliant with the BioPAX or KGML data formats. We will add in BiP software framework a KEGG downloader module to directly download the last version of KEGG pathways, making it possible to perform pathway enrichment analysis by using pathway data coded in KGML. BiP [33] is freely available, and we are working to make the updated version of BiP available as soon as possible. Thus, updated pathway information avoids to underrate the functional significance of relevant genes, preventing the lost of some possible significant results. A consideration that could explain the different pathway enrichment results obtained by CePa and SPIA is due to the use of an outdated version of KEGG (containing only 108 pathways). Whereas BiP and pathDIP used the last version of KEGG containing 139 pathways (at the time of writing), allowing them to enrich more pathways and providing more consistent pathways enrichment concerning the investigated cancer genes list.

## Discussion

To better understand the impact of the PEA on a gene or protein list of interest, we performed PEA employing the three couple of gene lists obtained from TCGA and GEO databases, respectively, related to the colorectal (CC), endometrial (EC), and thyroid (TC) cancers. The goal of PEA was to evaluate the capability of BiP to identify relevant pathways for the three types of cancer under investigation with respect to the other tools.

For the CC genes list and KEGG database, BiP enriched 274 pathways from COAD data set, and 53 pathways from GSE41011 with a *p* value $\le 0.005$. BiP enriched *insulin signaling pathway* (*p* value = $9.53\times 10^{-12}$), *Notch signaling pathway* (*p* value = $1.68\times 10^{-05}$), and *apoptosis* (*p* value = 0.002), all of which are well recognized pathways whose dysregulation can contribute in accelerate the risk of CC development and progression. It is worth to note that, in the intersection between the top 10 enriched pathway from both COAD and GSE41011 data sets, only BiP identified as novel candidate risk pathways the *Metabolic pathway* (*p* value = $4.33\times 10^{-152}$) which was disregarded by the other tools, the *Transcriptional misregulation in cancer* (*p* value = $2.44\times 10^{-28}$), and *proteoglycans in cancer* (*p* value = $3.08\times 10^{-21}$) pathways, which were ignored by CePa and SPIA tools. The enrichment of the three pathways related to CC is also described in [128, 129]. Zhao et al. [129] illustrates a protocol to figure out some critical biomarkers associated with colorectal cancer. The authors obtain the CC differential expressed genes from the GEO database (GSE32323 data set), whereas the KEGG pathway database was used to enrich those genes. [128] shows a pipeline to investigate possible competing endogenous RNA (ceRNA) networks in CC. The authors obtain the CC differential expressed genes from the TCGA database, whereas the KEGG pathway database was used to enrich those genes. The overlap between the pathway enrichment results obtained by BiP and those in [128, 129] confirms the effectiveness of BiP in identifying pathways that play a relevant role in complex diseases.

For the EC genes list and KEGG database, BiP enriched 276 pathways from UCEC data set, and 252 pathways from GSE63678 data set with a (*p* value $\le 0.005$). BiP enriched *p53 signaling pathway* (*p* value = $2.16\times 10^{-08}$), *VEGF signaling pathway* (*p* value = $8.60\times 10^{-05}$), and *Ras signaling pathway* (*p* value = $1.56\times 10^{-09}$), all of which are well recognized pathways whose dysregulation can contribute in EC development. Noticeably, in the intersection between the top 10 enriched pathway from both data sets UCEC and GSE63678, only BIP identified as novel candidate risk pathways *Metabolic pathway* (*p* value = $3.59\times 10^{-107}$), *MAPK signaling pathway* (*p* value = $4.41\times 10^{-31}$) in endometrial cancer (see [130], and [131]), which were ignored by the other tools.

For the TC genes list and KEGG database, BiP enriched 280 pathways from THCA data set, and 54 pathways from GSE65144 with a ( *p* value $\le 0.005$). BiP enriched *Thyroid hormone pathway* (*p* value = $1.35\times 10^{-06}$), *Rap1 signaling pathway* (*p* value = $1.65\times 10^{-08}$), and *Parathyroid hormone synthesis, secretion and action pathway* (*p* value = $1.56\times 10^{-09}$), all of which are well recognized pathways whose dysregulation can contribute to TC development. Noticeably, in the intersection between the top 10 enriched pathways from both data sets UCEC and GSE63678, only BIP identified as novel candidate risk pathways *Ubiquitin mediated proteolysis pathway* (*p* value = $1.75\times 10^{-17}$) and *Oxidative phosphorylation pathway* (*p* value = $7.49\times 10^{-17}$) and *reticulum pathway*. [132–134] and [95] describe the involvement of those pathways in TC, which were ignored by the other tools.

Finally, we compared BiP enrichment results with those obtained by Yang et al. [135] by using the same gene lists and pathway database. It is worthy to note that BiP enriches the same relevant pathways of Yang et al. This result confirms the effectiveness of BiP in identifying pertinent pathways in the condition under investigation, even if different data sets related to the same disease, are used. The production of very different pathway enrichment results for each investigated dataset associated with the same disease may limit the usefulness of those results in clinical and therapeutic scenarios. The production of very different pathway enrichment results for each investigated dataset associated with the same disease may limit the usefulness of those results in clinical and therapeutic scenarios. We used the Jaccard and meet-min indices to better prove the stability and the similarity between the enrichment results obtained from each couple of cancer datasets. The obtained results show that BiP provides more stable enrichment results than other tools, when analyzing different genes or proteins data sets related to the same diseases.

We would highlight the key role of the *metabolic pathway* in reprogramming cancer cells, that might play an important role in the progress of several types of cancers. Studies on cancer metabolism show that metabolic pathways aberrations and reprogramming are necessary to sustain rapid cell proliferation, tumor progression and cell death resistance [136]. Cancer metabolic reprogramming represents a fundamental trait of most cancer cells. Several works have evidenced that this metabolic reprogramming is an active process governed by oncogenes and cancer suppressors, which provides energy to the cancer cells [137]. Indeed, La Vecchia et al. in [138] provide a review of key findings in cancer metabolism pathway as a significant contributor of tumor initiation, growth, and metastatic dissemination in CC. Also, in [139] many findings show that metabolic pathway contributes to endometrial cancer cell survival and tumor growth are provided. Finally, in [140] a recent molecular study in thyroid cancer revealed that oncogenes and tumor suppressor genes not only control growth and apoptotic phenotypes of thyroid carcinomas but also directly affect the outcome of treatment and or disease progression in thyroid cancer. Thus, it appears clear that metabolic pathway dysregulation is a crucial factor in regulating tumor progression and survival in all the investigated tumor types. Thus, the association of this pathway with all three cancers types in our study is not surprising and confirms the effectiveness of BiP in identifying pathways that play a relevant role in complex diseases.

To the best of our knowledge, we were not able to find any evidence in literature proving the involvement of *Branched chain amino acid catabolism pathway* in developing, sustaining or growing EC. In [141] Sweatt et al. describe the implication of *Branched chain amino acid catabolism pathway* to regulate anabolic signals in digestive systems, as well as in neurons of the peripheral nervous system. To the best of our knowledge, we were not able to find any evidence in literature proving the involvement of *Removal of DNA patch containing a basic residue pathway* with EC. We didn’t get any results even looking for the *Removal of DNA patch containing a basic residue pathway* in KEGG. This result point outs that CePa to perform pathway enrichment used an older version of KEGG, that impact negatively on the enrichment results, since it provide an obsolete pathway name. In the current version of KEGG the *Removal of DNA patch containing a basic residue pathway* may have been replaced by the *DNA base excision repair pathway*. Furthermore, this can contribute to explain why even using different gene lists related to different cancer types, CePa provides the same first 8 pathway in both CC and EC enrichment results. Notability, the number of genes in common among the CC and EC cancer data sets is equal to 30; value that does not justify this overlap in the pathways enrichment results using different genes data sets. We also investigated the *neuroactive ligand receptor interaction pathway* that is a collection of receptors located on the plasma membranes, involved in the transduction of signals from the extracellular environment into cells [142]. The *neuroactive ligand-receptor interaction pathway* contains a very large gene set, consisting of more than 300 genes representing a variety of signalling molecules including many types of neuroreceptors. Many of these neuroreceptor genes have been found to be associated with multiple cancers, as well as playing an important role in the development of CC [143, 144]. Thus, the association of this gene set with CC observed in our study is not surprising, and confirms the effectiveness of BiP in identifying pathways that play a key role in complex diseases.

## Conclusion

In this work, we presented a comparative study of pathway enrichment analysis tools, conducted by using BiP, CePa, pathDIP and SPIA software tools. We have shown that the used database version influences the pathway enrichment results; the updated versions do not underrate the functional significance of relevant genes and proteins and do not omit the significant effects. This conclusion was specifically pronounced for CePa and SPIA because they are topology-based enrichment approaches and consequently expected to be most sensitive to the definition of a pathway available in the adopted database. At the same time, we observed that using an updated version of pathway databases (BiP) or an integrative pathway approach (pathDIP) led to more biologically consistent results.

The results obtained by BiP show good performance in terms of the number of relevant enriched pathways, especially compared to SPIA. BiP shows higher Jaccard and meet-min indexes values in almost all similarity comparisons results obtained from different gene lists of the same cancer types than CePa, pathDIP, and SPIA, suggesting BiP’s efficacy to achieve more reliable results. Of course, part of the boost in performance may be due to using more recently update versions of Reactome and KEGG databases, considering the difference in the size of the two databases.

As future works, we are improving the enrichment approach to limit the number of enriched pathways, ensuring at the same time the best possible result accuracy and improving their effectiveness. Finally, we will extend the BiP parsing capability to make it compatible with as many pathway representation formats as possible, such as SBML, GMTL, and XML-like.

## Supplementary Information

**Additional file 1.** Pathway Enrichment Analysisuser guide illustrating how to download genes or proteins data setsfrom GEO and TCGA databases and highlighting how to use thedownloaded data sets with the surveyed PEA software tools.

**Additional file 2.** The list of relevant enriched pathways(*p* value $\le 0.005$) obtained by BiP using each cancergene list and the related intersection between the two lists ofenriched pathways referring to the same cancer type.

**Additional file 3.** The list of relevant enriched pathways(*p* value $\le 0.005$) obtained by CePa using each cancergene list and the related intersection between the two lists ofenriched pathways referring to the same cancer type.

**Additional file 4.** The list of relevant enriched pathways(*p* value $\le 0.005$) obtained by pathDip using eachcancer gene list and the related intersection between the two listsof enriched pathways referring to the same cancer type.

**Additional file 5.** The list of relevant enriched pathways(*p* value $\le 0.005$) obtained by SPIA using each cancergene list and the related intersection between the two lists ofenriched pathways referring to the same cancer type.

## Data Availability

The datasets used and analyzed in this study are freely available in *TCGA* and *GEO*. *TCGA* datasets links: https://portal.gdc.cancer.gov/projects/TCGA-COAD, https://portal.gdc.cancer.gov/projects/TCGA-THCA, https://portal.gdc.cancer.gov/projects/TCGA-UCEC. *GEO* datasets links: https://www.ncbi.nlm.nih.gov/geo/query/acc.cgi?acc=GSE41011, https://www.ncbi.nlm.nih.gov/geo/query/acc.cgi?acc=GSE65144, https://www.ncbi.nlm.nih.gov/geo/query/acc.cgi?acc=GSE63678. Reactome database link: https://reactome.org/download-data, KEGG database link: https://www.kegg.jp, BiP software tool link: https://gitlab.com/giuseppeagapito/bip, CePa software tool link: http://cran.r-project.org/web/packages/CePa/, pathDIP software tool link: http://ophid.utoronto.ca/pathDIP, SPIA software tool link: http://bioconductor.org/packages/SPIA/. Also, all the links to the datasets and materials have been provided through the manuscript.

## Abbreviations

API: Application Program Interface
BiP: BioPAX-Parser
CC: Colorectal cancer
CePa: Centrality-based Pathway Enrichment
CNA: Copy-number alteration
DEG: Differential expressed gene
EC: Endometrial cancer
FDR: False discovery rate
GEO: Gene Expression Omnibus
GSEA: Gene Set Enrichment Analysis
GWAS: Genome-Wide Association Studies
HT: High Throughput
JI: Jaccard similarity Index
KEGG: Kyoto Encyclopaedia of Gene and Genome
mi: meet-min index
miRNA: micro RNA
NGS: Next Generation Sequencing
ORA: Over Represented Analysis
PEA: Pathway enrichment analysis
SNPs: Single nucleotide polymorphism
SPIA: Signaling Pathway Impact Analysis
TC: Thyroid cancer
TCGA: The Cancer Genome Atlas
TEA: Topological Enrichment Analysis
