# Supplementary material for: Using BioPAX-Parser (BiP) to enrich lists of genes or proteins with pathway data
Source: BMC Bioinformatics. 2021 Sep 30;22(Suppl 13):376. doi: 10.1186/s12859-021-04297-z (PMC8482563; doi:10.1186/s12859-021-04297-z)
Supplement: Supplementary file 1 — Additional file 1. Pathway Enrichment Analysisuser guide illustrating how to download genes or proteins data setsfrom GEO and TCGA databases and highlighting how to use thedownloaded data sets with the surveyed PEA software tools. [file 12859_2021_4297_MOESM1_ESM.pdf]

RESEARCH

Pathway Enrichment Analysis User Guide

Giuseppe Agapito[1,3*] and Mario Cannataro[2,3]

*Correspondence: agapito@unicz.it
[1]Department of Legal, Economic and Social Sciences, University "Magna Graecia", Catanzaro, Italy
Full list of author information is available at the end of the article

To perform pathway enrichment analysis, the users need internet connection, a pathway enrichment analysis framework, Java, R, and a web-browser installed on his/her computer.

- To obtain genes differentially expressed from microarray data, users can use GEO (`https://www.ncbi.nlm.nih.gov/geo`), a web-repository that collects several microarray data sets for many different diseases.
- To obtain human cancer samples to identify relevant genomic changes that may play a role in cancer development, users can use TCGA (`https://www.cancer.gov/about-nci/organization/ccg/research/structural-genomics/tcga`), a web repository that collects several types of human cancer, including nine rare tumors.
- To perform pathway enrichment analysis, users could use BiP, CePa, pathDIP, or SPIA.

Data Sets Download

GEO data sets download

G.1 Upon connecting to GEO, the user will input the disease of choice in the Search box, paying attention to select GEODataset from the drop-down menu locate at the left of the Search box. As second step, clicking Search the number of founded items will be visualized. Clicking on them will open the search results page.

G.2 It is then possible to filter the results according to the researcher interest, making it easier to find the data set user is looking for.

G.3 At the information data set page, clicking "Analyze with GEO2R" will open the page needed to obtain the differential genes.

G.4 In GEO2R page, the user will need to first set the groups to use in the analysis, clicking on *define groups*. The user will then select the appropriate samples and link them to their group.

G.5 Click on "Analyze" button located at the bottom to run the differential gene expression analysis.

G.6 Top results are shown in the table at the bottom of the page. Selecting "Download full table" to obtain the results.

The main steps listed above are shown in Figure 1.

TCGA data sets download

T.1 Upon connecting to Genomic Data Commons Portal (https://portal.gdc.cancer.gov), the user will input the disease he/she is looking for in the Search box, or by clicking on the human vignette situated on the right corner. As a result, the Explorer page will be open.

T.2 From the Explorer page, it is then possible to download the genes list selecting the Gene tab.
T.3 At the cBioPortal (https://www.cbioportal.org) web page, user can annotate gene lists (if available) by selecting the data set of interest from those available listed in the main page. After selecting the annotation data set, it is then possible to click the "Query By Gene" button located at the bottom of the page. Clicking "Query By Gene" will open the query building page.
T.4 In the Query building page, user can paste into the text area the previous downloaded gene lists to annotate, then click "Submit Query".
T.5 The results are shown in the table at the bottom of the page. Select "Download full table" to obtain the results.

The main steps listed above are shown in Figure 2.

Enrichment

The gene lists obtained from the previous steps are going to be used to perform pathway enrichment analysis (PEA) by using an enrichment tool.

BiP

To perform PEA by using BiP, user must launch BiP and then load the genes or proteins list, and selecting the pathway database to compute the enrichment. Gene list can contain Gene Symbols, or UniProt IDs. User can choose if using any downloaded pathway data in BioPAX format for the analysis. Results will be visualized in a tabular format, that will be saved in a Comma Separated Value (CSV) or txt file. A more detailed vignette of the full BiP analysis capabilities is available at `https://gitlab.com/giuseppeagapito/bip`.

CePa

To perfom PEA by using CePa it is necessary to write a simple R script. User must run R, load CePa package and then load the gene list, using the "read.csv" command. Gene list can contain Gene Symbols, or UniProt IDs. The pathway enrichment analysis can be performed by using the "cepa.all()" function. CePa will use the embedded KEGG pathway database to compute the enrichment. In the following we show a simple R script to compute pathway enrichment by using CePa. CePa is available at `http://cran.r-project.org/web/packages/CePa/`.

```
library("CePa")
#read the disease-genes input file
genes <- read.csv("/genes.txt", sep = "\t")
colnames(genes) <- "list" #add the name to the column
res = cepa.all(dif = gene.list$dif) #run the PEA analysis
plot(res) #display the PEA results
```

pathDip

To perform PEA by using pathDIP, user must connect to the pathDip web-site and then paste the gene list into the Search box. Gene list can contain Gene Symbols,

Entrez Gene IDs or UniProt IDs. It is important to choose the correct pathway sources to use for the analysis. Before run the analysis, user can choose if download or visualize the results. If the user chooses to download the results, they will be included in a txt file. A more detailed vignette of the full pathDip analysis capabilities is available at http://ophid.utoronto.ca/pathDIP/.

SPIA
To perform PEA by using SPIA user must run R, load SPIA package and then write the R scripts. User must load the gene list containing Enterez IDs, using the "read.csv()" command. The pathway enrichment analysis is executed through the "spia()" function. Following we show a simple R script to compute pathway enrichment by using SPIA. SPIA is available at http://bioconductor.org/packages/SPIA/.

```
library(SPIA)
#read the disease-genes input
df <- read.csv("/dataset.txt", sep = "\t", header = TRUE)
decolon <- df$log2FC
#add the name to the column
names(decolon) <- as.vector(df$enterez)
allcolen <- df$enterez
#run the PEA analysis
res <- spia(de=decolon, all=allcolen, organism ="hsa", nB=2000)
plot(res) #display the PEA results
```

The enriched pathways can be used by researchers to give a biological meaning to huge lists of genes proteins of interest detached from their biological context, making easier to use into clinical and therapeutic scenarios.

Acknowledgements
This work is the extended version of "Using BioPAX-Parser (BiP) to annotate lists of biological entities with pathway data" accepted at the "1st International Workshop on Conceptual Modeling for Life Sciences (CMLS 2020)", in conjunction with the "39th International Conference on Conceptual Modeling (ER 2020)", and published on the Lecture Notes on Computer Science (LNCS 12584) proceedings, Springer Nature.

Funding
Not applicable.

Availability of data and materials
TCGA database link:
https://www.cancer.gov/about-nci/organization/ccg/research/structural-genomics/tcga
GEO database link: https://www.ncbi.nlm.nih.gov/geo
Reactome database link : https://reactome.org/download-data
KEGG database link: https://www.kegg.jp
BiP software tool link: https://gitlab.com/giuseppeagapito/bip
CePa software tool link: http://cran.r-project.org/web/packages/CePa/
pathDIP software tool link: http://ophid.utoronto.ca/pathDIP
SPIA software tool link: http://bioconductor.org/packages/SPIA/
Also, all the links to the datasets and materials have been provided through the manuscript.

Ethics approval and consent to participate
No ethics approval was required for the study.

Consent for publication
Not Applicable.

Competing interests
The authors declare that they have no competing interests.

Author details
[1]Department of Legal, Economic and Social Sciences, University "Magna Graecia", Catanzaro, Italy. [2]Department of Medical and Surgical Sciences, University "Magna Graecia", Catanzaro, Italy. [3]Data Analytics Research Center, University "Magna Graecia", Catanzaro, Italy.

References

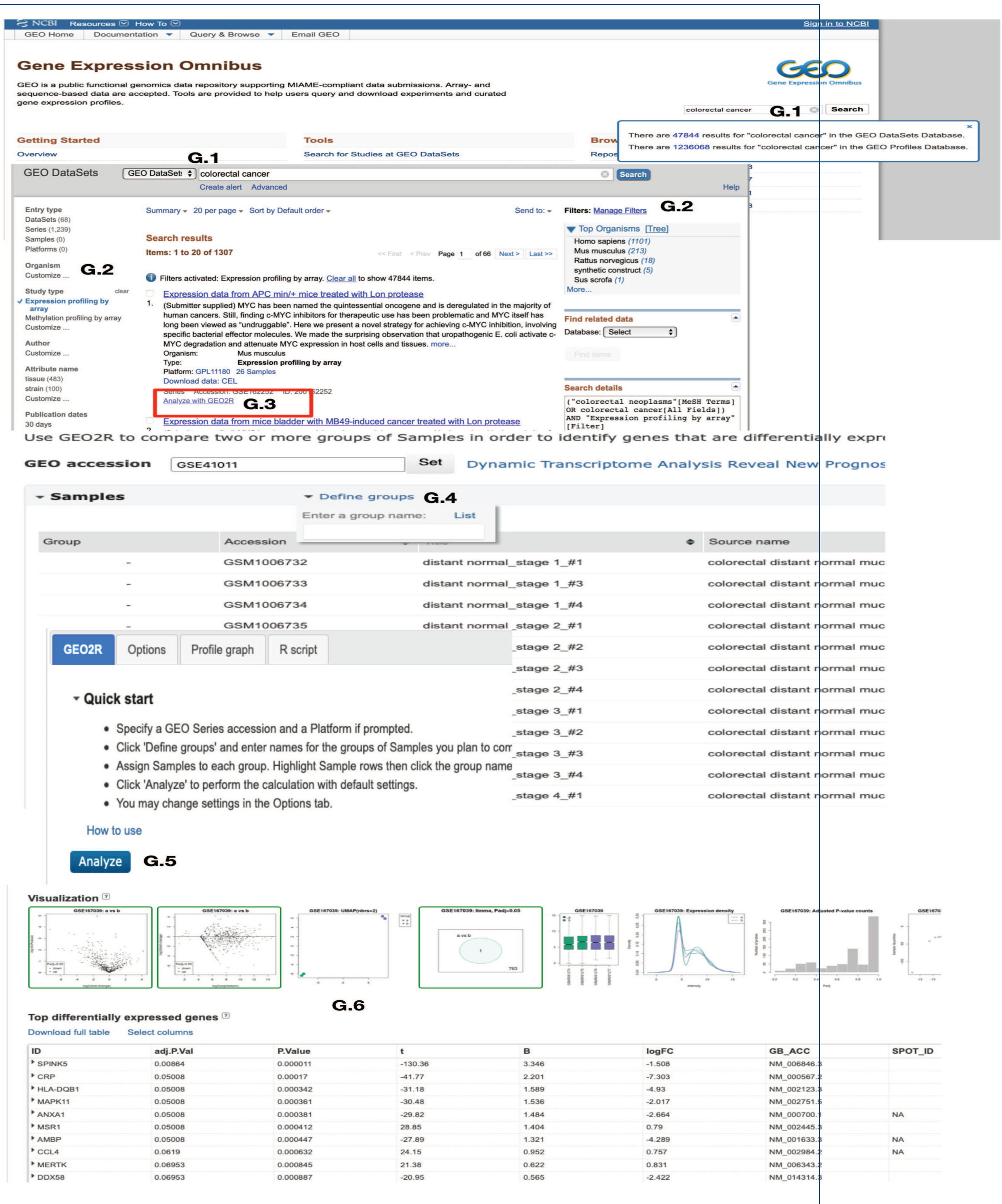

Figure 1 GEO data sets download user guide.

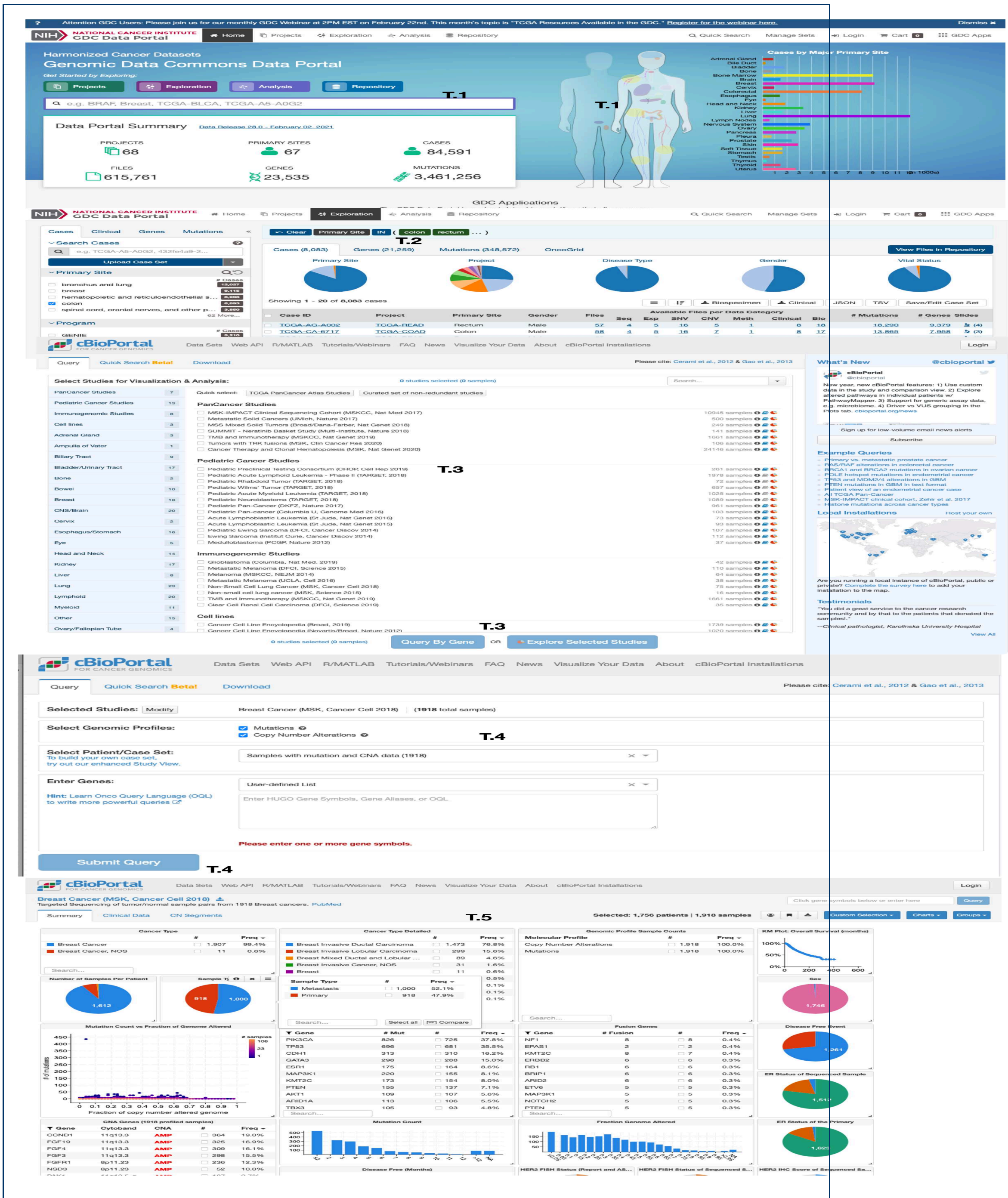

Figure 2 GEO data sets download user guide.
